# Research on energy management strategies for high-power diesel-electric hybrid tractors based on double deep Q-network

**DOI:** 10.1038/s41598-025-02044-5

**Published:** 2025-05-17

**Authors:** Yiwei Wu, Yifan Zhao, Changkai Wen, Junjiang Zhang, Xianghai Yan

**Affiliations:** 1 College of Vehicle and Traffic Engineering, Henan University of Science and Technology, Luoyang, 471003 China; 2 State Key Laboratory of Intelligent Agricultural Power Equipment, Luoyang, 471039 China; 3 College of Engineering, China Agricultural University, Beijing, 100083 China

**Keywords:** Deep reinforcement learning, Hybrid tractor, Energy management strategy, Hardware in the loop, Engineering, Mechanical engineering

## Abstract

Addressing the issues of complex parameter tuning and challenging online application of control algorithms for energy management strategies in current hybrid tractors. Taking high-power diesel-electric hybrid tractor as the research object, dynamic models for the tractor under three operating conditions—plowing, rotary tillage, and transportation—were established. With engine power as the agent’s control action, drive motor power and battery SOC as state variables, and tractor equivalent fuel consumption and battery SOC as the reward function, an energy management strategy based on deep reinforcement learning was designed using the double deep Q-network (DDQN) algorithm. Finally, a hardware-in-the-loop (HIL) testing platform was set up to conduct HIL tests on the designed tractor model and energy management strategy. The test results demonstrate that, in comparison to the power-following-based energy management strategy, the DDQN strategy: under plowing conditions, achieves a 1.18% savings in SOC, reduces equivalent fuel consumption by 10.40%, and decreases diesel consumption by 4.59%; under rotary tillage conditions, consumes an additional 3.09% of SOC, but reduces equivalent fuel consumption by 9.78% and diesel consumption by 4.77%; and under transportation conditions, consumes an additional 1.49% of SOC, reduces equivalent fuel consumption by 9.57%, and increases diesel consumption by 0.65%.

## Introduction

### Aims and motivation

In the face of increasingly severe energy shortages and environmental pollution issues, the demand for energy-saving and eco-friendly agricultural machinery in modern agricultural production has become increasingly urgent^1,2^. Tractors are the core power machinery in agricultural production activities. Currently, most mainstream agricultural tractors are powered by diesel engines, which suffer from issues such as low fuel efficiency and high carbon emissions^3,4^. With the successful application of hybrid technology in the passenger vehicle sector, hybrid tractors have also become an important research direction in the current development of tractor technology^5,6^. In the face of complex and varied farmland operation scenarios, hybrid tractors, compared to fuel-powered tractors, are equipped with multiple power sources. By rationally allocating the power output among different power sources, the engine can operate in an optimal state according to the workload conditions, reducing fuel consumption while avoiding the range limitations of pure electric tractors^7–9^. Therefore, the application research of hybrid technology in the field of tractors is of great significance to the advancement of agricultural machinery technology and agricultural sustainability^10,11^.

Energy management strategies have a direct impact on the power performance and fuel economy of hybrid power systems and are key technologies for achieving energy-efficient control in hybrid electric tractors^12,13^. With the advancement of computer technology and artificial intelligence, deep reinforcement learning (DRL) has begun to be actively applied in the research of hybrid power energy management strategies^14^. Energy management strategies based on DRL enable continuous learning through interactions between the agent and the simulation environment, allowing for the ongoing updating of control parameters to achieve optimal control, and they exhibit excellent adaptability to complex operating conditions^15,16^. This paper analyzes the fuel economy model of hybrid electric tractors and the load characteristics of their operating conditions, and designs an energy management strategy based on DRL using the DDQN algorithm. Finally, HIL testing was conducted on the constructed tractor model and energy management strategy model to verify the effectiveness of the control results obtained using the DDQN strategy.

### Literature review

At the current stage, energy management strategies are mainly classified into the following three types: rule-based, optimization-based, and learning-based methods^17^. The design principle of rule-based energy management strategies is relatively simple and has been well-established in the application of hybrid vehicles^18^. Zou et al.^19^ designed an energy management strategy for fuel cell hybrid vehicles that combines power-following and fuzzy control, improving the vehicle’s economy. Wei et al.^20^ proposed a fuzzy control energy management strategy based on driving pattern recognition using a long short-term memory neural network, which reduced fuel consumption by 5.8% compared to traditional fuzzy control strategies. Wang et al.^21^ utilized a genetic algorithm to optimize an energy management strategy based on fuzzy control, while implementing driving condition recognition based on the K-means clustering method, effectively enhancing fuel economy. However, rule-based energy management strategies rely on design experience for development, making it relatively difficult to debug the control strategy and achieve optimal fuel economy^22^.

Optimization-based energy management strategies involve designing corresponding cost functions and constraints based on vehicle simulation parameters, and then solving them using various optimization algorithms to achieve optimal control^23^. Curiel-Olivares et al.^24^ designed an energy management strategy based on model predictive control, which reduced fuel consumption by 7.2% compared to rule-based energy management strategies. Zhang et al.^25^ designed an energy management strategy based on the dynamic programming algorithm for series–parallel hybrid tractors, effectively improving the tractor’s fuel efficiency. Cheng et al.^26^ proposed an energy management strategy based on the gain power-to-fuel coefficient, enhancing the fuel-saving efficiency of hybrid vehicles. However, the control effectiveness of energy management strategies based on optimization relies on specific operating conditions, and the algorithms involve significant computational complexity, making it difficult to apply in real-time and online^27^.

Compared to rule-based and optimization-based energy management strategies, learning-based energy management strategies do not rely on developers’ expert experience for rule design, nor do they depend on extensive calculations conducted through the establishment of precise mathematical models of hybrid vehicles to determine optimal control. Instead, they utilize learning-based data processing methods and the accumulation of experience from the vehicle’s current and past operational state parameters to predict the vehicle’s future operational state. This involves repeating the process of experience accumulation and continuously updating control parameters through trial and error to ascertain the optimal control strategy for hybrid vehicles. Such strategies offer advantages such as being model-free and capable of real-time autonomous learning of optimal strategies^28,29^.

Li et al.^30^ designed an energy management strategy based on deep Q-learning for hybrid battery electric vehicles, effectively reducing energy loss. Ye et al.^31^ combined an energy management strategy based on DRL with digital twin technology, significantly improving the energy efficiency of automobiles. Hu et al.^32^ designed an energy management strategy based on DRL, which improved the fuel economy of vehicles compared to rule-based energy management strategies. Currently, research on DRL-based energy management strategies mostly focuses on passenger vehicles, with relatively limited applications in the field of hybrid tractors. The operating conditions of tractors in field work are more complex and variable compared to passenger cars. Therefore, this paper designs an energy management strategy based on DRL using the DDQN algorithm, incorporating three main operating conditions of tractors. This provides a comprehensive reference for the application of DRL in energy management strategies for hybrid tractors. Chen et al.^33^ developed an energy management strategy based on DRL using the Deep Deterministic Policy Gradient algorithm, which demonstrated stronger adaptability to complex driving conditions. Qi et al.^34^ dynamically adjusted the reward function of the control strategy using weight coefficients obtained through inverse reinforcement learning, achieving favorable fuel-saving effects. Xu et al.^35^ proposed a supervised learning-based method for driving cycle pattern recognition, which could accurately predict road conditions and improve the fuel economy of hybrid vehicles. Wu et al.^36^ introduced an energy management strategy based on the Deep Deterministic Policy Gradient algorithm, offering performance close to global optimal dynamic programming and enabling optimal energy distribution for vehicles in continuous spaces. Wang et al.^37^ integrated computer vision with DRL, utilizing visual information captured by onboard cameras to enable the algorithm to autonomously learn optimal control strategies, reducing fuel consumption and achieving 96.5% of the performance of global optimal dynamic programming. Finally, the research content of the references mentioned in this paper is summarized in Table 1, categorized by research object, control strategy, consideration of multiple operating conditions simultaneously, and whether HIL or real-vehicle validation was conducted.

**Table 1:** A summary of the literature.

Refs.	Research object		Energy management strategy			Operating condition		Validation
	Automobile	Tractor	Rule based	Optimisation based	Learning based	Multi condition	Single condition	
^19^	✓		✓				✓	✗
^20^	✓		✓				✓	✗
^21^	✓		✓			✓		✗
^24^		✓		✓			✓	✗
^25^		✓		✓			✓	✗
^26^	✓			✓		✓		✓
^30^	✓				✓	✓		✗
^31^	✓				✓		✓	✓
^32^	✓				✓	✓		✗
^33^	✓				✓	✓		✗
^34^	✓				✓	✓		✓
^35^	✓				✓	✓		✗
^36^	✓				✓	✓		✗
^37^	✓				✓	✓		✓

### Research gaps and contributions

As shown in Table 1, current research on energy management strategies for hybrid vehicles is predominantly focused on passenger vehicles, with limited studies in the field of hybrid electric tractors. Moreover, existing research on hybrid electric tractors mainly revolves around rule-based and optimization-based strategies, leaving a significant gap in the application of DRL to hybrid electric tractors. Furthermore, the field operation conditions of tractors are more complex and variable compared to those of passenger cars. Therefore, during the process of researching and designing energy management strategies, it is of great significance to simultaneously analyze and discuss the three typical operating conditions of tractors, namely plowing, rotary tillage, and transportation, and ultimately test and verify the control effectiveness of the energy management strategy using HIL testing technology, for the robustness and practicality of the energy management strategy.

Hence, to address the aforementioned research gaps, this paper designs an energy management strategy based on DRL, utilizing the DDQN algorithm and incorporating the three main operating conditions of tractors. This study provides a comprehensive reference for the application of DRL in energy management strategies for hybrid electric tractors. The main research contributions of this paper are as follows:

1. Considering the research characteristics of operating conditions and fuel economy for hybrid electric tractors, we simultaneously analyzed and established dynamic models for three typical operating conditions of tractors: plowing, rotary tillage, and transportation.
2. Based on the DDQN algorithm, we constructed a DRL-based energy management strategy for hybrid electric tractors, with engine power as the agent’s control action, drive motor power and battery SOC as the state variables, and tractor equivalent fuel consumption and battery SOC as the reward function.
3. We investigated the control effectiveness of the DRL energy management strategy under different operating conditions of tractors.
4. A HIL testing platform was built based on the control principle of the energy management strategy to verify the effectiveness of the control strategy.

### Paper organization

The main research contents of this paper are as follows. In “Introduction” section explains the main structure and system parameters of the series-connected high-power diesel-electric hybrid tractor. In “Structure of the power system” section constructs dynamic models for the tractor under three operating conditions: plowing, rotary tillage, and transportation. In “Hybrid tractor model construction” section proposes a DRL energy management strategy based on the DDQN algorithm and designs an energy management strategy based on power following (PF) as a comparative strategy. In “Energy management strategy design” section conducts HIL testing on the control effectiveness of the proposed strategy by building an HIL test platform, effectively validating the control performance of the energy management strategy. In “Analysis and discussion of HIL test results” section summarizes the research content and test results.

## Structure of the power system

**Fig. 1:**
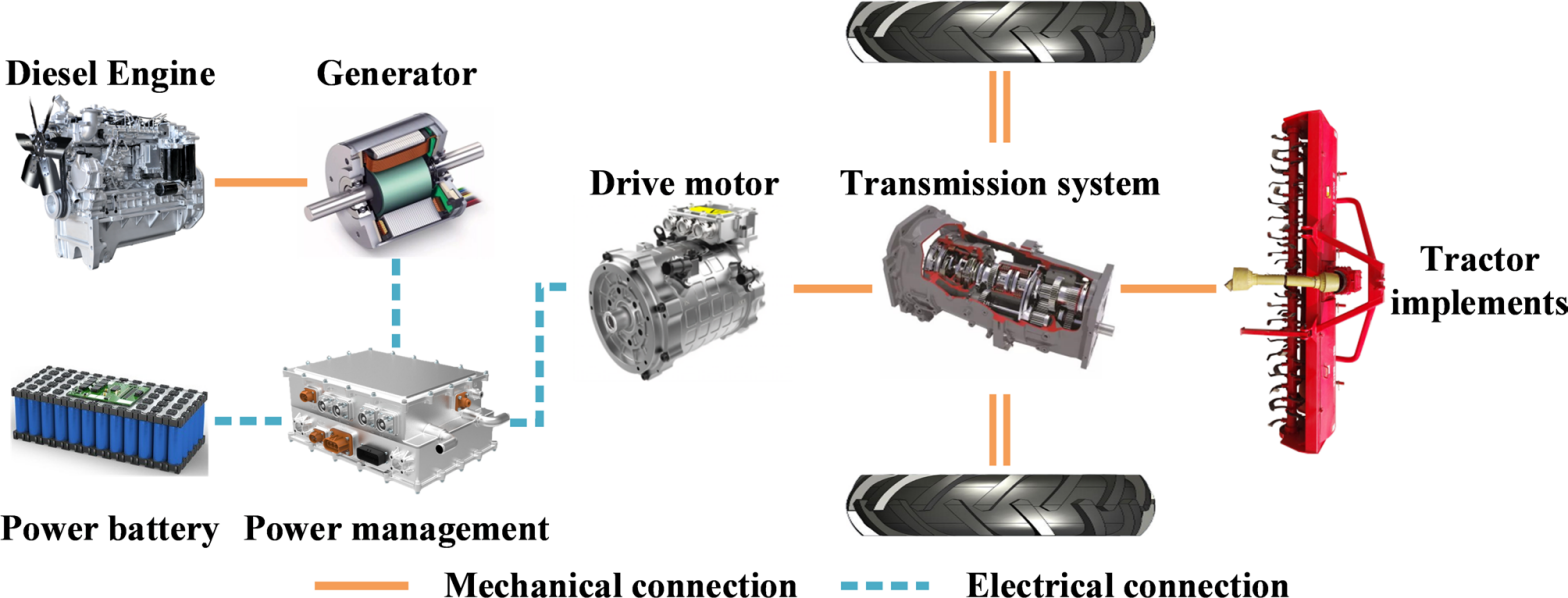
Structure of tractor power system.

This paper focuses on a series hybrid tractor as the research object, and the specific structure of the tractor’s power system is shown in Fig. 1. The main components include a diesel engine, a generator, a traction battery, power management, a drive motor, a transmission system, and tractor implements. The working mode of the hybrid power system is as follows: When the traction battery has sufficient charge, it supplies power to the drive motor, which then drives the tractor and its operational implements through the transmission system. When the battery charge is insufficient, the diesel engine starts up and simultaneously powers the generator to recharge the traction battery. The main parameters of the tractor power system are shown in Table 2, including specific details such as the main performance parameters of the diesel engine and drive motor, as well as the overall vehicle parameters of the tractor and the rated capacity of the traction battery.

**Table 2:** Parameters of power system components.

Component	Parameter	Value (unit)
Diesel engine	Rated power	85 (kW)
	Rated speed	2300 (r/min)
	Maximum torque speed	1500–1700 (r/min)
Drive motor	Rated power	63 (kW)
	Peak power	125 (kW)
	Rated speed	2000 (r/min)
	Rated torque	300 (N m)
Tractor parameters	Tractor mass	1905 (kg)
	Wheel radius	0.3556 (m)
	Frontal area	2.95 (m^2^)
Power battery	Rated capacity	70 (A h)
	Rated voltage	330 (V)
	SOC	0.25–0.90

## Hybrid tractor model construction

### Driver model

The driver model is based on the PI control principle, which calculates the throttle and brake pedal opening during tractor operation according to the difference between the target vehicle speed and the actual vehicle speed through PI control. The modeling principle is shown in the following equation:

1 $$k_{\alpha } = k_{p} e + k_{i} \int {edt}$$

2 $$\left\{ {\begin{array}{*{20}l} {k_{ac} = k_{\alpha } ,} \hfill & {\quad k_{\alpha } \in \left( {0,1} \right)} \hfill \\ {k_{br} = k_{\alpha } ,} \hfill & {\quad k_{\alpha } \in \left( { - 1,0} \right)} \hfill \\ \end{array} } \right.$$

3 $$e = v_{ref} - v_{act}$$

where *k*_*α*_ is the pedal opening, *k*_*p*_ is the proportional coefficient, *k*_*i*_ is the integral coefficient; *e* is the difference between the desired velocity of the tractor and the current velocity of the tractor, km/h; *k*_*ac*_ is the accelerator pedal opening, and *k*_*br*_ is the brake pedal opening; *v*_*ref*_ is the desired velocity of the tractor, km/h, and *v*_*act*_ is the current velocity of the tractor, km/h.

### Drive motor model

**Fig. 2:**
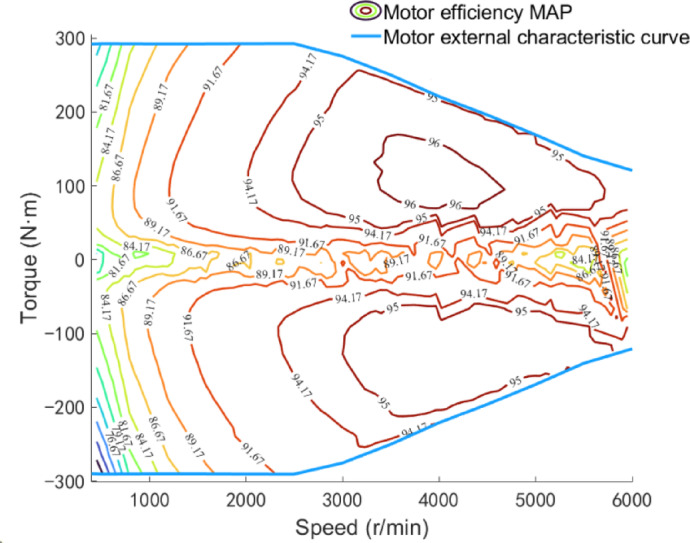
Motor efficiency MAP.

Numerical modeling of the drive motor is conducted based on data interpolation, as shown in Fig. 2. Using bench test data, the efficiency MAP and external characteristic curve of the drive motor varying with speed and torque are fitted^38^. Based on the external characteristic curve of the drive motor, the maximum torque at the current motor speed is obtained, and the power of the drive motor is controlled through the pedal opening output by the driver model. The modeling principle is shown in the following equation:

4 $$P_{m} = \frac{{n_{m} T_{m} }}{{9549\eta_{m} }}$$

5 $$T_{m} = k_{ac} T_{m\_\max } \left( {n_{m} } \right)$$

6 $$\eta_{m} = f\left( {n_{m} ,T_{m} } \right)$$

where *P*_*m*_ is the drive motor power, kW; *n*_*m*_ is the drive motor speed, r/min; *T*_*m*_ is the drive motor torque, N m; *η*_*m*_ is the drive motor efficiency; *T*_*m*_max_ is the maximum torque at the current drive motor speed.

### Transmission system model

In the powertrain architecture of a series hybrid tractor, the power supplied by the drive motor is transmitted through the transmission system to the driving wheels and tractor implements^39^. The rotational speed of the drive motor can also be calculated based on the actual vehicle speed of the tractor and the parameters of the transmission system. The modeling principle is illustrated by the following equation:

7 $$F_{tr} = \frac{{T_{m} i_{g} i_{0} \eta_{T} }}{{R_{w} }} - F_{br}$$

8 $$F_{br} = k_{br} F_{br\_\max }$$

9 $$n_{m} = \frac{{v_{act} i_{g} i_{0} }}{{0.377R_{w} }}$$

where *F*_*tr*_ is the forward traction force acting on the tractor through the transmission system by the drive motor torque, N; *i*_*g*_ is the gear ratio of the transmission; *i*_0_ is the gear ratio of the final drive; *η*_*T*_ is the efficiency of the transmission system; *R*_*w*_ is the radius of the drive wheel, m; *F*_*br*_ is the braking force of the brake, N; *F*_*br*_max_ is the maximum braking force of the brake, N.

### Tractor plowing condition dynamics model

Under plowing conditions, the driving force of a tractor is primarily influenced by the plowing resistance and rolling resistance. The balance between the driving force and the driving resistance is illustrated by the following equation:

10 $$F_{t} = F_{tr} - \left( {F_{L} + F_{f} } \right)$$

11 $$F_{L} = Zbhk$$

12 $$F_{f} = mgf\cos \omega$$

The actual speed of the tractor is calculated based on its current driving force.

13 $$v_{act} = \int {\frac{{F_{t} }}{3.6m}} dt$$

where *F*_*t*_ is the driving force, N; *F*_*L*_ is the plowing resistance, N; *F*_*f*_ is the rolling resistance, N; *Z* is the number of plowshares; *b* is the width of a single plowshare, cm; *h* is the plowing depth, cm; *k* is the specific resistance of the soil, N/cm^2^; *m* is the operating mass of the tractor, kg; *g* is the acceleration of gravity, m/s^2^; *f* is the rolling resistance coefficient; *ω* is the slope angle, (°).

### Tractor rotary tillage condition dynamics model

Under the condition of rotary tillage, the balance of driving resistance for tractors is relatively complex. Therefore, power coupling is adopted to model the rotary tillage condition. The operating power of the tractor during rotary tillage is mainly determined by the traveling power and the rotary tiller power. The modeling principle is shown in the following equation^40^:

14 $$P_{m} = P_{drive} + P_{r}$$

15 $$P_{drive} = \frac{{v_{act} \left( {F_{f} + F_{i} } \right)}}{{3600\eta_{T} }}$$

16 $$F_{i} = mg\sin \alpha$$

17 $$P_{r} = \frac{{3.6kBhv_{act} }}{{\eta_{r} }}$$

where *P*_*drive*_ is the tractor’s driving power, kW; *F*_*i*_ is the slope resistance, N; *P*_*r*_ is the power of the rotary tiller, kW; *B* is the width of the rotary tillage area, m; *η*_*r*_ is the transmission efficiency of the rotary tiller unit.

### Tractor transportation condition dynamics model

Under transportation conditions, the driving resistance of a tractor needs to take into account both acceleration resistance and air resistance. The balance relationship is shown in the following equation:

18 $$F_{t} = F_{tr} - \left( {F_{f} + F_{i} + F_{ac} + F_{ar} } \right)$$

19 $$F_{ac} = m\delta a$$

20 $$F_{ar} = \frac{{C_{D} Av_{act}^{2} }}{21.15}$$

where *F*_*ac*_ is the acceleration resistance, N; *F*_*ar*_ is the air resistance, N; *δ* is the tractor mass conversion coefficient; *a* is the tractor acceleration, m/s^2^; *C*_*D*_ is the wind resistance coefficient of the tractor; *A* is the windward area of the tractor, m^2^.

### Diesel engine and generator model

In the structure of a series hybrid power system, the engine and generator are not mechanically connected to the tractor’s powertrain. Therefore, the engine and generator are considered as a whole, and numerical modeling of the engine is performed based on engine bench test data^41^. The modeling principle is shown in the following equation:

21 $$P_{e} = \frac{{n_{e} T_{e} }}{9549}$$

22 $$P_{G} = P_{e} \eta_{G}$$

where *P*_*e*_ is the engine power, W; *n*_*e*_ is the engine speed, r/min; *T*_*e*_ is the engine torque, N m; *P*_*G*_ is the generator set power, W; *η*_*G*_ is the generator efficiency.

**Fig. 3:**
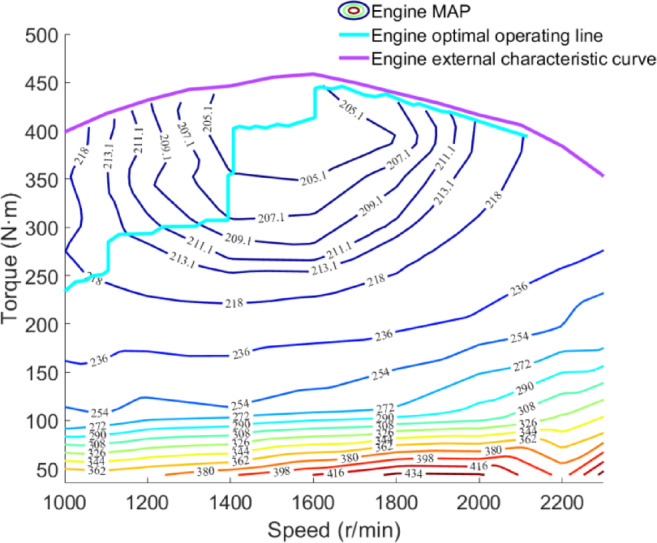
Universal characteristics of diesel engines.

As shown in Fig. 3, the engine’s universal characteristic data is fitted, including the engine fuel consumption rate MAP, the engine’s outer characteristic curve, and the engine’s optimal operating line (OOL).

Hybrid tractors have two energy sources: electricity and fuel. To balance the energy consumption costs of these two sources, equivalent fuel consumption is used as an evaluation metric, calculated as shown in the following equation^42^:

23 $$fuel\left( t \right) = \int_{0}^{{t_{f} }} {\left( {E\left( t \right) + \frac{{j_{m} P_{B} \left( t \right)}}{{j_{c} \eta_{B} }}} \right)} dt$$

24 $$E\left( t \right) = \frac{{P_{e} b_{e} }}{{1000 \times 3600 \times \rho_{f} }}$$

25 $$b_{e} = f\left( {n_{e} ,T_{e} } \right)$$

where *fuel*(*t*) is the equivalent fuel consumption, L; $t_{f}$ is the simulation termination time; *E*(*t*) is the fuel consumption at time t, L; *j*_*m*_ is the electricity price, CNY/kWh; *P*_*B*_(*t*) is the battery power at time *t*, kW; *j*_*c*_ is the fuel price, CNY/L; *b*_*e*_ is the engine fuel consumption rate, g/kWh; *ρ*_*f*_ is the diesel density, g/L.

### Power battery model

**Fig. 4:**
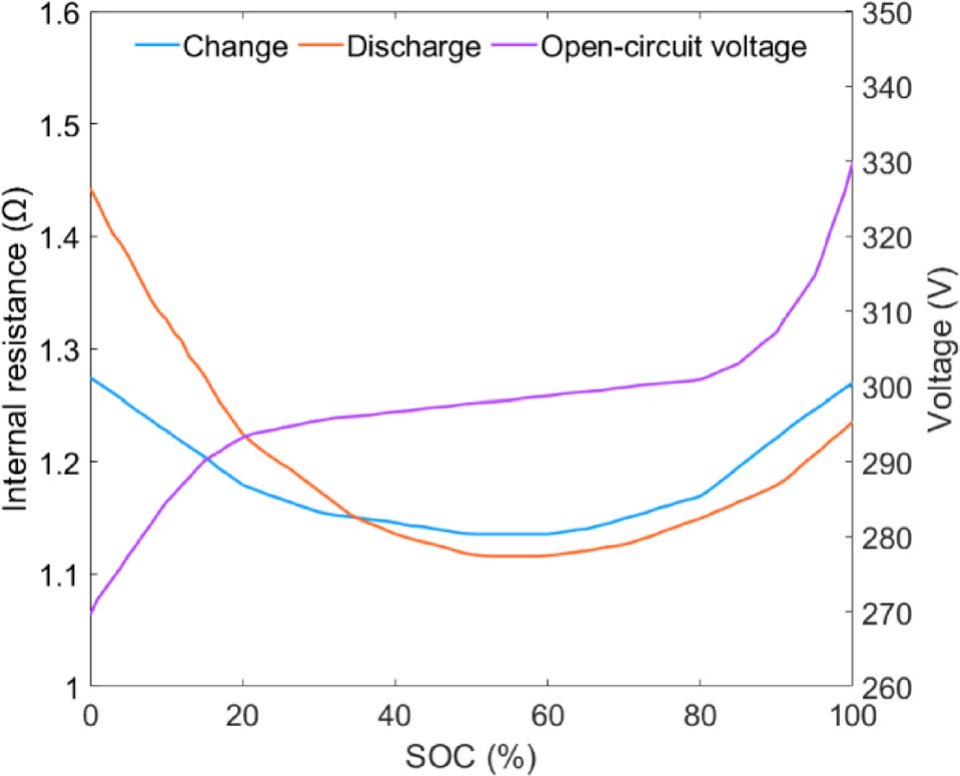
Charging and discharging characteristics of power batteries.

The equivalent circuit internal resistance model is used to model the power battery, ignoring the influence of factors such as temperature and battery life on the SOC^43^. As shown in Fig. 4, the charging and discharging characteristics of the power battery are presented, including the internal resistance change curve and the open-circuit voltage change curve during battery charging and discharging. The modeling principle is shown in the following equation:

26 $$\mathop {{\text{SOC}}}\limits^{ \bullet } = - \frac{{U_{oc} - \sqrt {U_{oc}^{2} - 4R_{{\text{int}}} P_{B} } }}{{2Q_{B} R_{{\text{int}}} }}$$

27 $$U_{oc} = f\left( {{\text{SOC}}} \right)$$

28 $$R_{{\text{int}}} = f\left( {{\text{SOC}}} \right)$$

29 $$P_{B} = \left\{ {\begin{array}{*{20}l} {\left( {P_{m} + P_{G} } \right)\eta_{B} ,} \hfill & {\quad \left( {P_{m} + P_{G} } \right) < 0} \hfill \\ {\frac{{\left( {P_{m} + P_{G} } \right)}}{{\eta_{B} }},} \hfill & {\quad \left( {P_{m} + P_{G} } \right) > 0} \hfill \\ \end{array} } \right.$$

where *U*_*oc*_ is the open-circuit voltage of the power battery, V; *R*_*int*_ is the internal resistance of the power battery, Ω; *Q*_*B*_ is the rated capacity of the battery, A h; *η*_*B*_ is the charge and discharge efficiency of the power battery. When (*P*_*m*_ + *P*_*e*_) is less than 0, the power battery is charging; when (*P*_*m*_ + *P*_*e*_) is greater than 0, the power battery is discharging.

### Simulation model of the whole series hybrid tractor

**Fig. 5:**
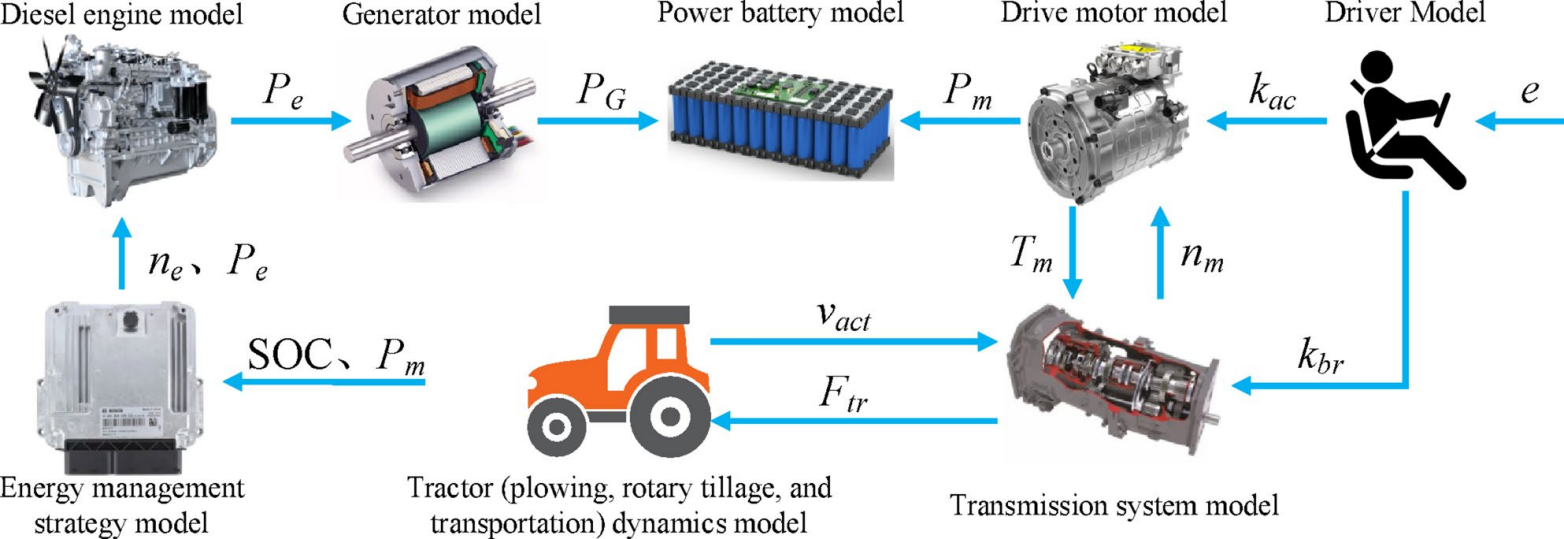
Tractor simulation model structure.

Modeling of the tractor is based on Matlab/Simulink, with the specific model structure shown in Fig. 5. The simulation calculation principle of the model is as follows: The driver model calculates the tractor’s pedal opening signals *k*_*ac*_ and *k*_*br*_ based on the difference *e* between the target and actual vehicle speeds. The drive motor model, transmission system model, and dynamics models of the tractor under various operating conditions calculate the current load power *P*_*m*_ of the drive motor based on the pedal opening signals. The energy management strategy model determines the operating state and output power *P*_*e*_ of the diesel engine model based on the tractor’s current battery SOC and drive motor power *P*_*m*_, using an energy management strategy, and then calculates the generator power *P*_*G*_ through the generator model. Finally, the battery model calculates the change in SOC based on the generator power *P*_*G*_ and drive motor power *P*_*m*_. The specific parameters of the tractor simulation model are shown in Table 3.

**Table 3:** Tractor simulation model parameters.

Parameter	Value	Unit	Parameter	Value	Unit
*Z*	3	–	*f*	0.12	–
*b*	25	cm	*B*	1.25	m
*h*	20	cm	*δ*	1.1	–
*k*	5	N/cm^2^	*C*_*D*_	0.32	–
*m*	2145	kg	*ρ*_*f*_	835	g/L
*j*_*m*_	0.58	CNY/kWh	*j*_*c*_	7.91	CNY/L

## Energy management strategy design

### Energy management strategy based on DDQN

#### DDQN energy management strategy model

Q-learning is a value-based reinforcement learning algorithm that, based on the Bellman Optimality Principle, learns to obtain the maximum cumulative reward through interactions between the agent and the environment. It iteratively updates the Q-value function to approximate the optimal control policy. The update rule for the Q-value function is shown in the following equation^44^:

30 $$Q\left( {s_{t} ,a_{t} } \right) \leftarrow Q\left( {s_{t} ,a_{t} } \right) + \alpha \left[ {r_{t} + \gamma \mathop {\max }\limits_{{_{{a_{t + 1} }} }} Q\left( {s_{t + 1} ,a_{t + 1} } \right) - Q\left( {s_{t} ,a_{t} } \right)} \right]$$

where *s* is the agent’s state; *a* is the agent’s action; *t* is the current time step; *Q*(*s*_*t*_, *a*_*t*_) is the Q-value of executing action at in state *s*_*t*_; *α* is the learning rate; *r*_*t*_ is the immediate reward; *γ* is the discount factor, which determines the present value of future rewards; $\mathop {\max }\nolimits_{{a_{t + 1} }} Q\left( {s_{t + 1} ,a_{t + 1} } \right)$ is the estimated maximum Q-value for all possible actions at time step *t* + 1.

Q-learning updates the selection of actions based on a greedy search strategy, as shown in Eq. (31). During the iterative update process, the value of *ε* gradually decreases, ultimately leading to the optimal control action strategy^45^.

31 $$a = \left\{ {\begin{array}{*{20}l} {a_{{\text{optimal }}} ,} \hfill & {\quad \left( {1 - \varepsilon } \right) \times 100\% } \hfill \\ {a_{{{\text{random}}}} ,} \hfill & {\quad \varepsilon \times 100\% } \hfill \\ \end{array} } \right.$$

where *ε* is the degree of greediness; *a*_*optimal*_ is the optimal action; *a*_*random*_ is the random action.

**Fig. 6:**
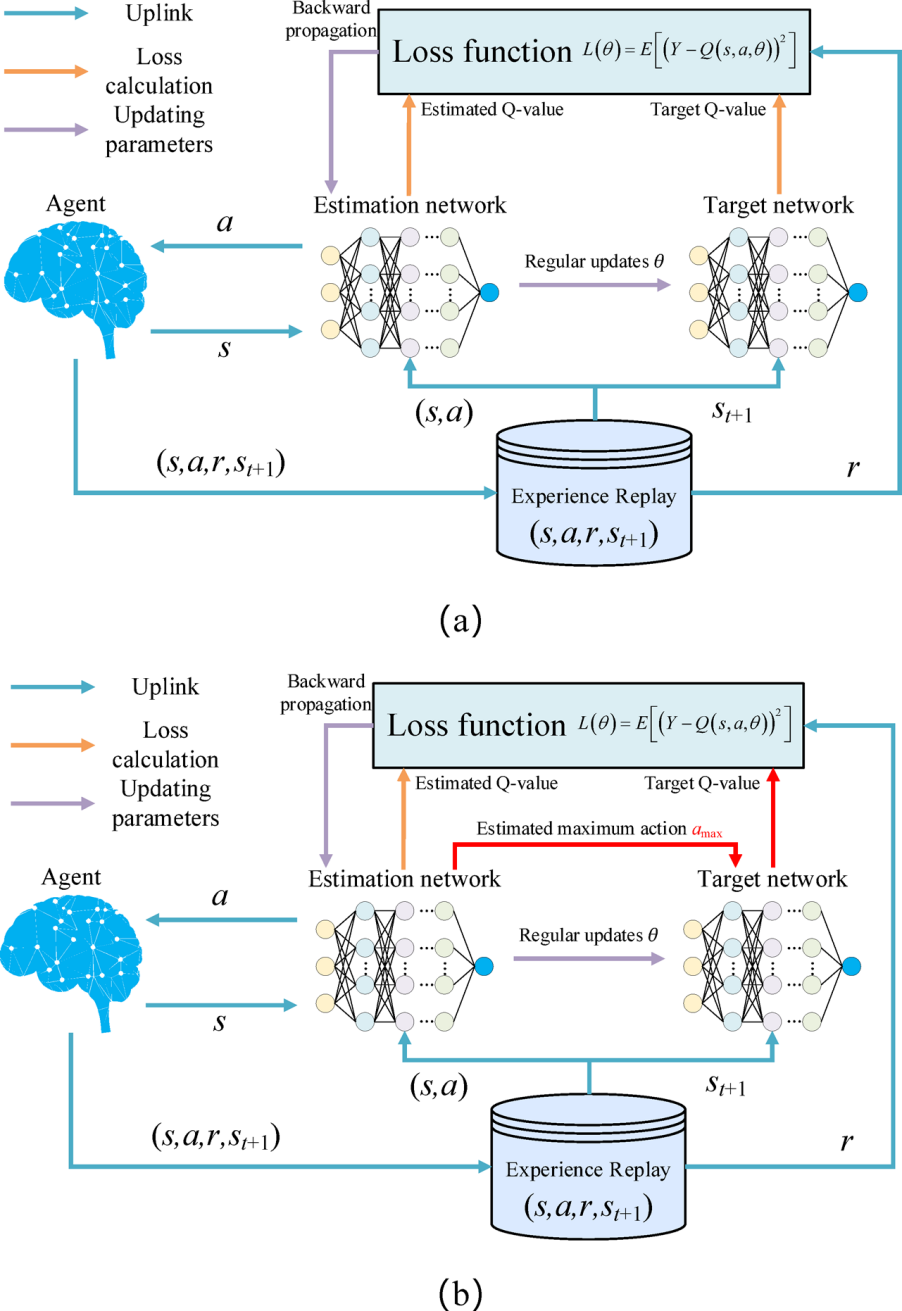
DRL algorithm architecture, (**a**) DQN, (**b**) DDQN.

Q-learning constructs a Q-value table to store the Q-values corresponding to all states and actions, and ultimately enables the agent to choose the action with the maximum reward based on these Q-values. However, when faced with high-dimensional and continuous state and action spaces, storing all states and actions based on a Q-value table becomes extremely difficult, often leading to the curse of dimensionality. The DRL algorithm, Deep Q-Network (DQN), introduces deep neural networks on the basis of Q-learning to address the functional dimension modeling issue of the Q-value table, effectively overcoming the dimensionality limitation of Q-learning in state spaces^46^. The structure of the DQN algorithm is illustrated in Fig. 6a. The DQN algorithm consists of two neural networks with identical structures but different parameters: the estimate network and the target network. The estimate network possesses the latest network parameters. After calculating the Q-value function at the current time step, the parameters of the target network are periodically updated. Finally, by defining a loss function, backpropagation is performed on both neural networks. Through training, the agent establishes a mapping relationship between the Q-value and the stateaction pair^47^.

By using a deep neural network to approximate the Q-value function, as shown in the equation below:

32 $$Q\left( {s_{t} ,a_{t} |\theta } \right) \approx Q\left( {s_{t} ,a_{t} } \right)$$

where *θ* is the neural network parameters.

The loss function of the DQN algorithm is defined as the difference between *Q*(*s*_*t*_, *a*_*t*_) and the discounted maximum Q-value of the next state *Q*(*s*_*t*+1_, *a*_*t*+1_), as shown in the equation below:

33 $$L\left( \theta \right) = \left[ {r_{t + 1} + \gamma \mathop {\max }\limits_{{_{{a_{t + 1} }} }} Q\left( {s_{t + 1} ,a_{t + 1} ,\theta_{t + 1} } \right) - Q\left( {s_{t} ,a_{t} ,\theta } \right)} \right]^{2}$$

Neural networks require training samples with high diversity. Therefore, the DQN algorithm introduces an experience replay mechanism. When updating the neural network parameters *θ*, a small subset of training samples is randomly selected from the experience pool, ensuring that the training samples maintain a certain level of independence throughout the neural network training process^48^. This is specifically shown in the following equation:

34 $$sample = \left( {s,a,r,s_{t + 1} } \right)$$

However, during the Q-value update process of the DQN algorithm, the estimation of the maximum Q-value for both the current state and the next state is based on the same Q-value function. This can lead to overestimation of the Q-values for some states and actions, which may be continually amplified during the iteration process, making it difficult for the algorithm to converge. The DDQN algorithm addresses the issue of overestimation of Q-values by introducing two Q-value functions to decouple action selection and action value evaluation^49^. The structure of the DDQN algorithm is illustrated in Fig. 6b. Instead of directly selecting the maximum Q-value from the target network, DDQN first selects the action corresponding to the maximum Q-value using the estimate network, as shown in the following equation:

35 $$a_{\max } = \mathop {\arg \max }\limits_{a} Q\left( {S_{t + 1} ,a,\theta } \right)$$

Then, the target network calculates the target Q-value based on the selected action amax. The calculation of the target Q-value *Y* and the loss function for DDQN is shown in the following equation:

36 $$Y = r_{t} + \gamma Q\left[ {s_{t + 1} ,a_{\max } ,\theta_{t + 1} } \right]$$

37 $$L\left( \theta \right) = E\left[ {\left( {Y - Q\left( {s,a,\theta } \right)} \right)^{2} } \right]$$

In the structure of a series hybrid power system, the changes in the SOC of the traction battery and the power of the drive motor can reflect the state changes and load variations of the tractor during operation. The operating state of the engine can determine the changes in SOC, and the ultimate goal of optimal control in energy management strategies is to reduce energy consumption. Therefore, the power of the drive motor and the SOC of the traction battery are taken as the state variables of the agent. The engine power is taken as the action variable of the agent and is discretized. Meanwhile, to further reduce energy consumption, the engine power is selected within the range of the OOL curve fitted in Fig. 3. The equivalent fuel consumption of the tractor and the SOC of the traction battery are used as the reward function, as specifically shown in the following equation:

38 $$S = \left\{ {{\text{SOC}}\left( t \right),P_{m} \left( t \right)} \right\}$$

39 $$A = \left\{ {P_{e1} ,P_{e2} ,P_{e3} , \ldots ,P_{en} } \right\},\quad A \in P_{e\_OOL}$$

40 $$r = - \left[ {\varphi fuel\left( t \right) + \beta \left( {{\text{SOC}}\left( t \right) - {\text{SOC}}_{0} } \right)^{2} } \right]$$

where *S* is the set of states; *A* is the set of actions; *P*_*e*_*OOL*_ is the optimal operating point of the engine on the OOL curve; *r* is the reward function; *φ* is the weighting factor for equivalent fuel consumption; *β* is the weighting factor for SOC change; SOC_0_ is the initial SOC of the power battery.

Additionally, to ensure that the DDQN algorithm can converge iteratively in a reasonable manner, the following constraints are added:

41 $$\left\{ {\begin{array}{*{20}l} {{\text{SOC}}_{\min } \le {\text{SOC}}\left( t \right) \le {\text{SOC}}_{\max } } \hfill \\ {P_{B\_\min } \le P_{B} \left( t \right) \le P_{B\_\max } } \hfill \\ {P_{e\_\min } \le P_{e} \left( t \right) \le P_{e\_\max } } \hfill \\ {n_{e\_\min } \le n_{e} \left( t \right) \le n_{e\_\max } } \hfill \\ {T_{e\_\min } \le T_{e} \left( t \right) \le T_{e\_\max } } \hfill \\ \end{array} } \right.$$

where SOC_min_ is the lower limit of SOC, and SOC_max_ is the upper limit of SOC; *P*_*B*_min_ and *P*_*B*_max_ are the minimum and maximum power of the power battery during operation; *P*_*e*_min_, *P*_*e*_max_, *n*_*e*_min_, *n*_*e*_max_, *T*_*e*_min_, and *T*_*e*_max_ are the minimum and maximum power, minimum and maximum speed, and minimum and maximum torque of the engine during operation, respectively.

#### Control process of the DDQN energy management strategy

**Fig. 7:**
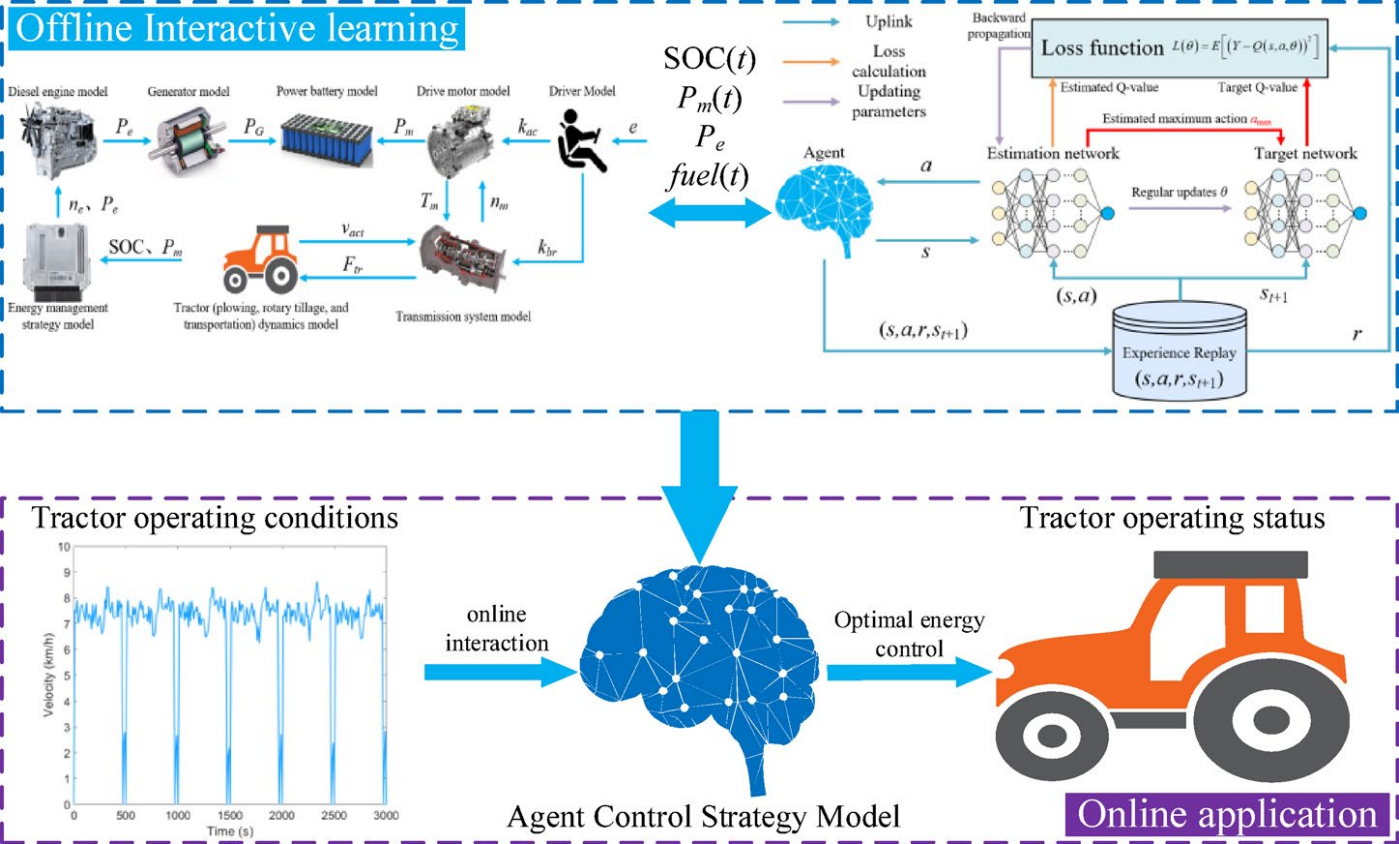
DDQN strategy control process.

The control flow of the DDQN strategy is illustrated in Fig. 7. The DDQN algorithm interacts offline with the tractor simulation model for learning. The agent learns the optimal control strategy through iterative training. Once the iteration converges, the agent updates and stores the control strategy parameters. During the online application phase of the control strategy, the agent interacts online with the tractor’s operating conditions based on the offline-learned optimal control strategy to determine the optimal control parameters for the tractor.

### Energy management strategy based on power following

**Fig. 8:**
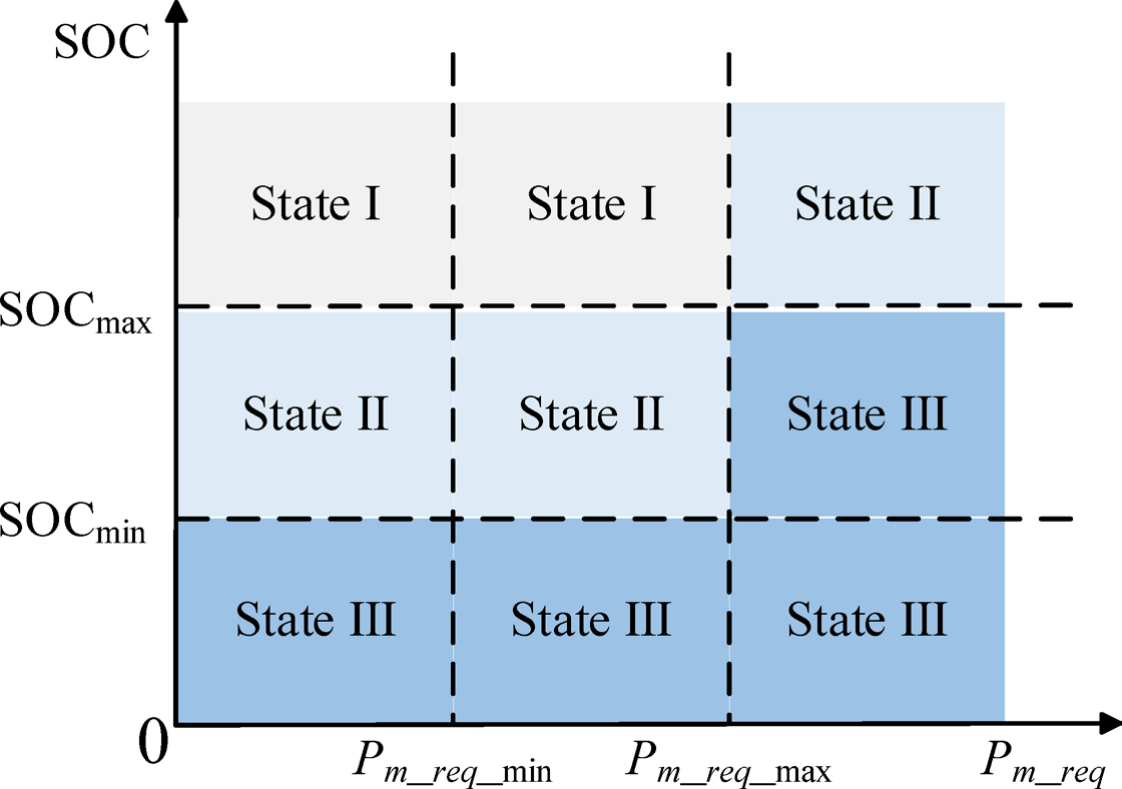
Power following schematic diagram.

The power-following energy management strategy is a deterministic rule-based energy management strategy^50^. In this study, it is used as a comparison strategy to analyze the control effectiveness of the DDQN strategy. In a series hybrid power system, the PF strategy controls the operating state of the engine based on the power of the drive motor and the SOC of the traction battery. The control rules of the PF strategy are illustrated in Fig. 8, and the specific rules are as follows:

- State I: When SOC ≤ SOC_min_, the engine starts.
- State II: When SOC_min_ ≤ SOC ≤ SOC_max_, if *P*_*m*_*req*_ ≥ *P*_*m*_*req*_max_, the engine starts immediately; otherwise, the engine maintains its previous start-stop state.
- State III: When SOC ≥ SOC_max_, if *P*_*m*_*req*_ ≥ *P*_*m*_*req*_max_, the engine maintains its previous start-stop state; otherwise, the engine shuts down.

To better compare the DDQN strategy with the PF strategy, this paper improves the PF strategy so that it can dynamically control the engine’s output power by following the trend of the drive motor power with a certain proportional coefficient. Simultaneously, like the DDQN strategy, the improved PF strategy can select the engine operating point within the range of the OOL curve fitted in Fig. 3, as shown in the following equation:

42 $$P_{e\_PF} = k_{PF} P_{m}$$

43 $$P_{e\_PF} \in P_{e\_OOL}$$

where *P*_*m*_*req*_ is the required power of the driving motor, *P*_*m*_*req*_min_ is the minimum required power of the driving motor, and *P*_*m*_*req*_max_ is the maximum required power of the driving motor; *P*_*e*_*PF*_ represents the engine power output by the PF strategy; *k*_*PF*_ is the proportional coefficient of the PF strategy for following the drive motor power.

## Analysis and discussion of HIL test results

### HIL test platform setup

**Fig. 9:**
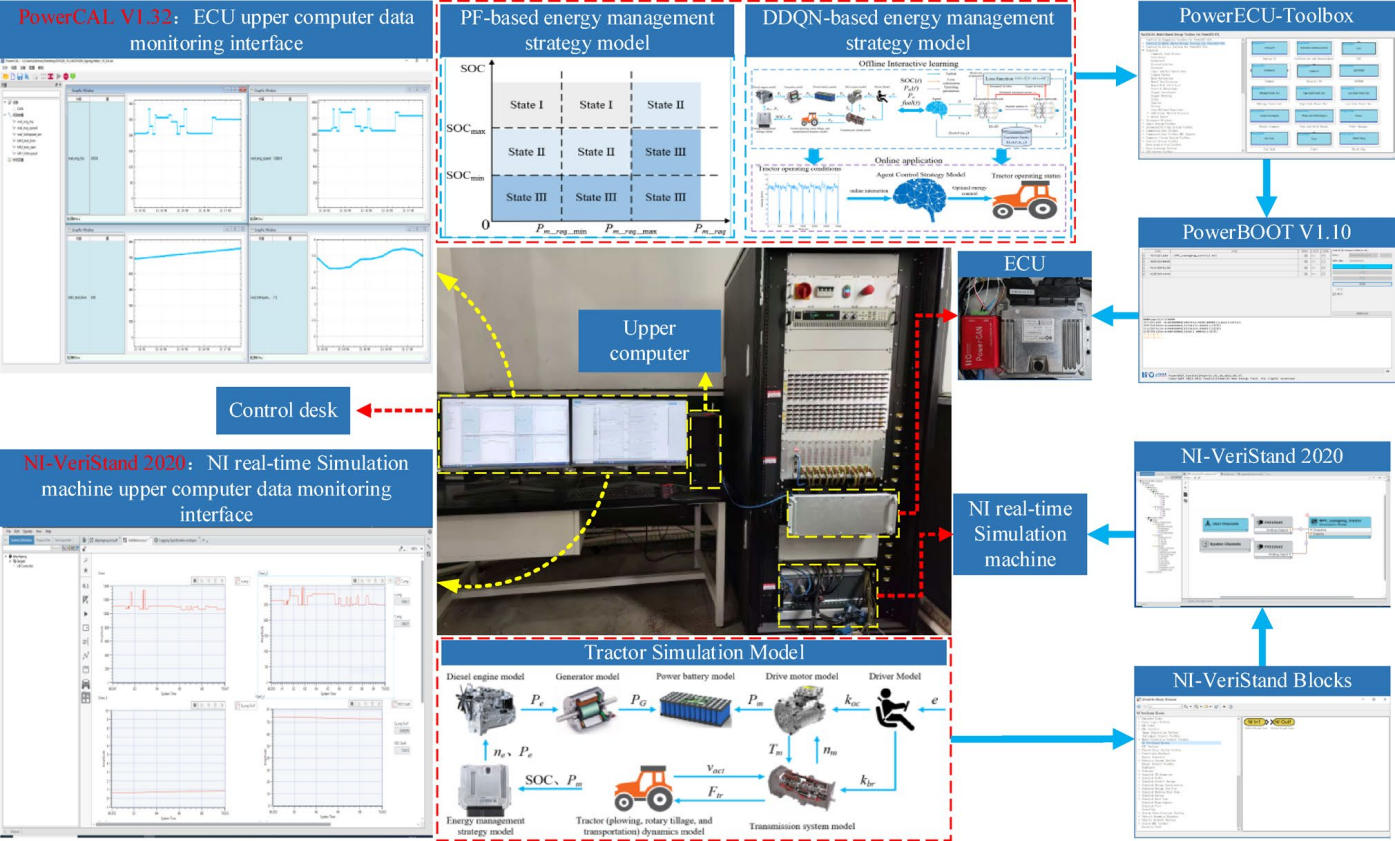
HIL test process.

To verify the effectiveness of the DDQN strategy, this paper has established a HIL testing platform as shown in Fig. 9 to conduct HIL tests on the proposed energy management strategy. The HIL testing platform is primarily composed of the vehicle ECU controller PowerECU-57A produced by Shandong Hydrogen Exploration New Energy Technology Co., Ltd. in China, and the HIL testing cabinet manufactured by National Instruments in the United States. The HIL testing process is illustrated in Fig. 9: The ECU communicates with the NI real-time simulation machine using analog voltage signals for data transmission. By utilizing the corresponding Matlab/Simulink plugins, PowerECU-Toolbox for the ECU and NI-VeriStand Blocks for the NI real-time simulation machine, appropriate analog I/O communication modules are added to the tractor model and energy management strategy model built in this paper. Using the target language compiler (TLC) files corresponding to the ECU and NI real-time simulation machine, along with the Matlab/RTW code generator, the tractor model and energy management strategy model are compiled into C code. The compiled code is then burned into the ECU and NI real-time simulation machine using PowerBOOT V1.10 and NI-VeriStand 2020, respectively. Finally, software configuration and data monitoring of the ECU and NI real-time simulator are performed using Power-CAL V1.32 and NI-VeriStand 2020.

### Result analysis

#### Plowing condition

**Fig. 10:**
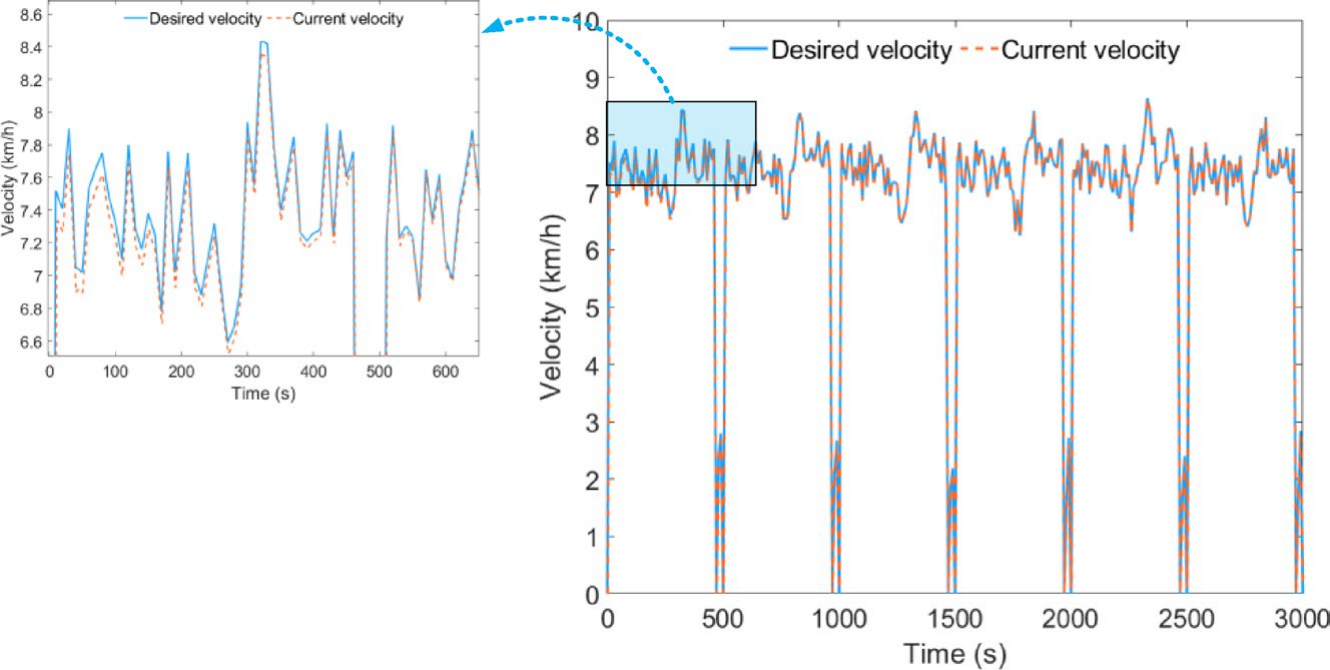
Vehicle velocity tracking effect under plowing condition.

Under plowing conditions, the desired vehicle velocity tracking performance during HIL testing is shown in Fig. 10. The average error between the desired vehicle velocity and the current vehicle velocity remains at 0.028 km/h, indicating that the simulation model can accurately exchange data during the HIL testing.

**Fig. 11:**
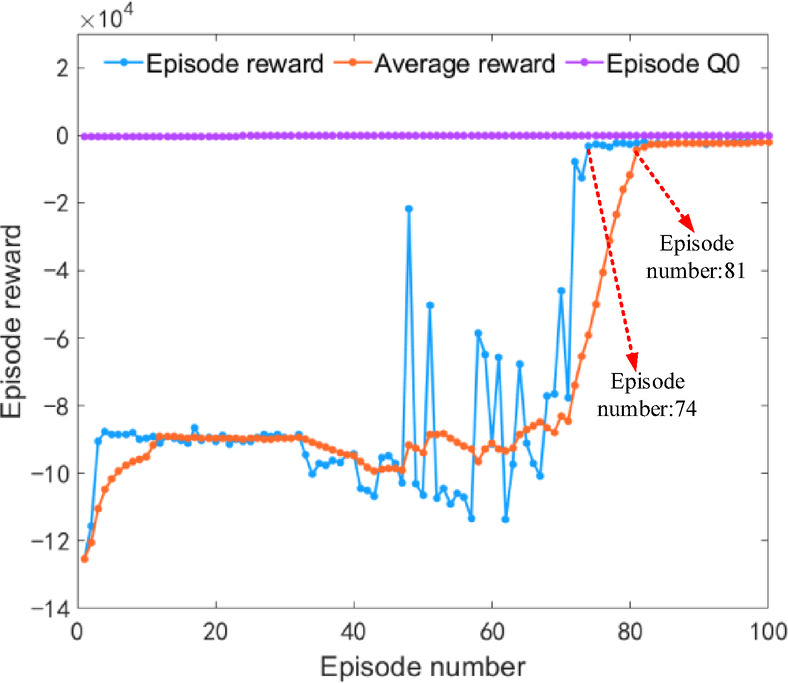
Iteration results of the DDQN algorithm under plowing condition.

Under plowing conditions, the iterative results of the DDQN algorithm are shown in Fig. 11. The iterative rewards and average iterative rewards begin to converge during the 74th and 81st iteration training processes, respectively. After 100 iterations of training, the reward curve tends to stabilize, verifying the reliability of the algorithm’s iterative results.

**Fig. 12:**
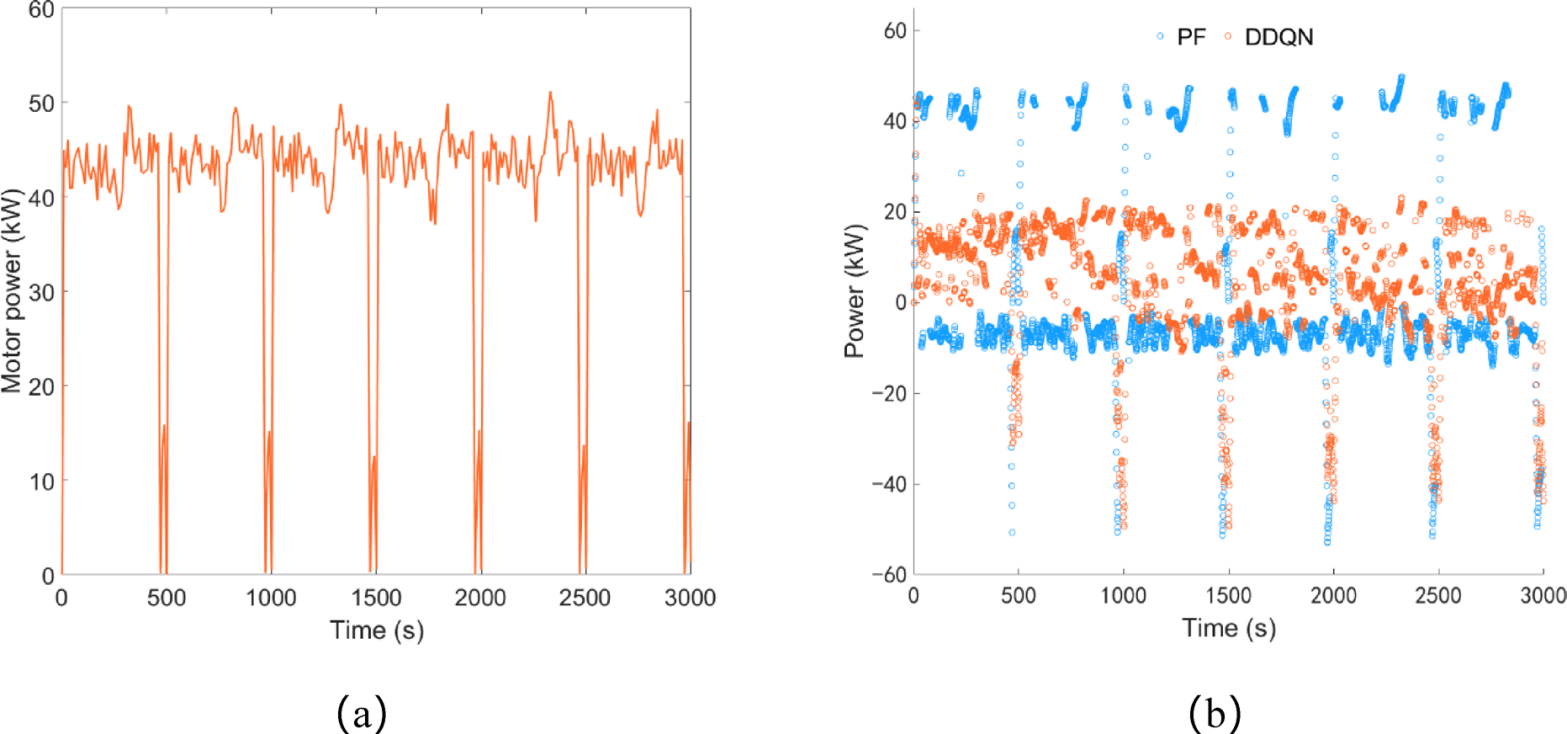
Plowing condition, (**a**) drive motor power; (**b**) battery power.

As shown in Fig. 12a, under the plowing condition, the driving motor power is concentrated between 40 and 50 kW, with an average load power of 40.90 kW and a total power consumption of approximately 34.09 kWh. As illustrated in Fig. 12b, under the control of the DDQN strategy, the engine starts up to charge the battery within approximately 15 s, and thereafter, the battery power remains below 23.44 kW. Under the control of the PF strategy, the battery power equals the driving motor power multiple times, indicating that during the PF strategy control process, the engine starts and stops multiple times to charge the battery.

**Fig. 13:**
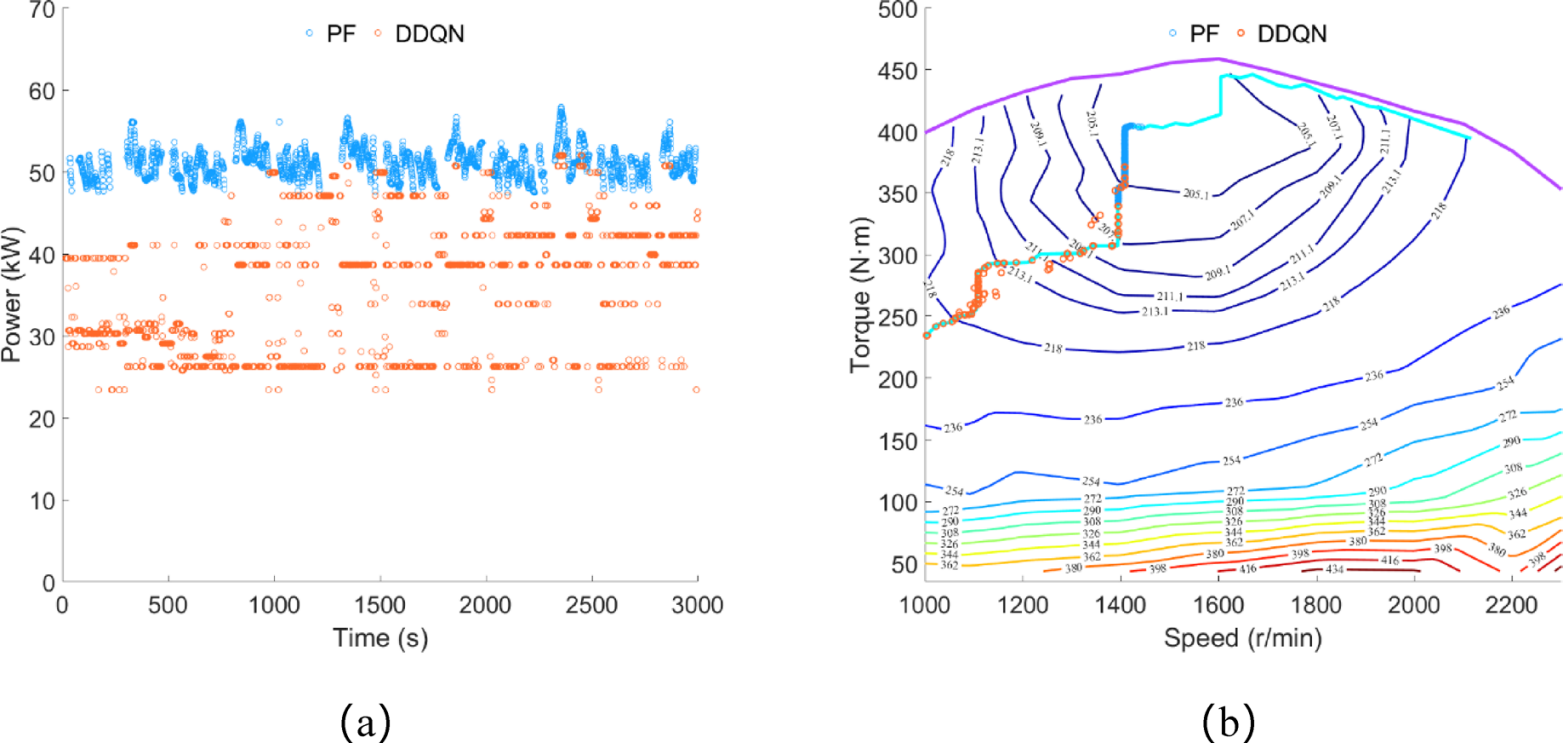
Plowing condition, (**a**) engine power; (**b**) engine operating point.

As shown in Fig. 13a, under the control of the DDQN strategy, the engine power is concentrated between 23 and 52 kW, with an average power of 35.62 kW and a total power consumption for electricity generation of approximately 29.70 kWh. Under the control of the PF strategy, the engine power is concentrated between 47 and 58 kW, with an average power of 36.19 kW and a total power consumption for electricity generation of approximately 30.17 kWh. As illustrated in Fig. 13b, the engine operating points selected by both the PF strategy and the DDQN strategy are concentrated near the OOL curve. However, under the control of the DDQN strategy, the engine’s operating range is wider.

**Fig. 14:**
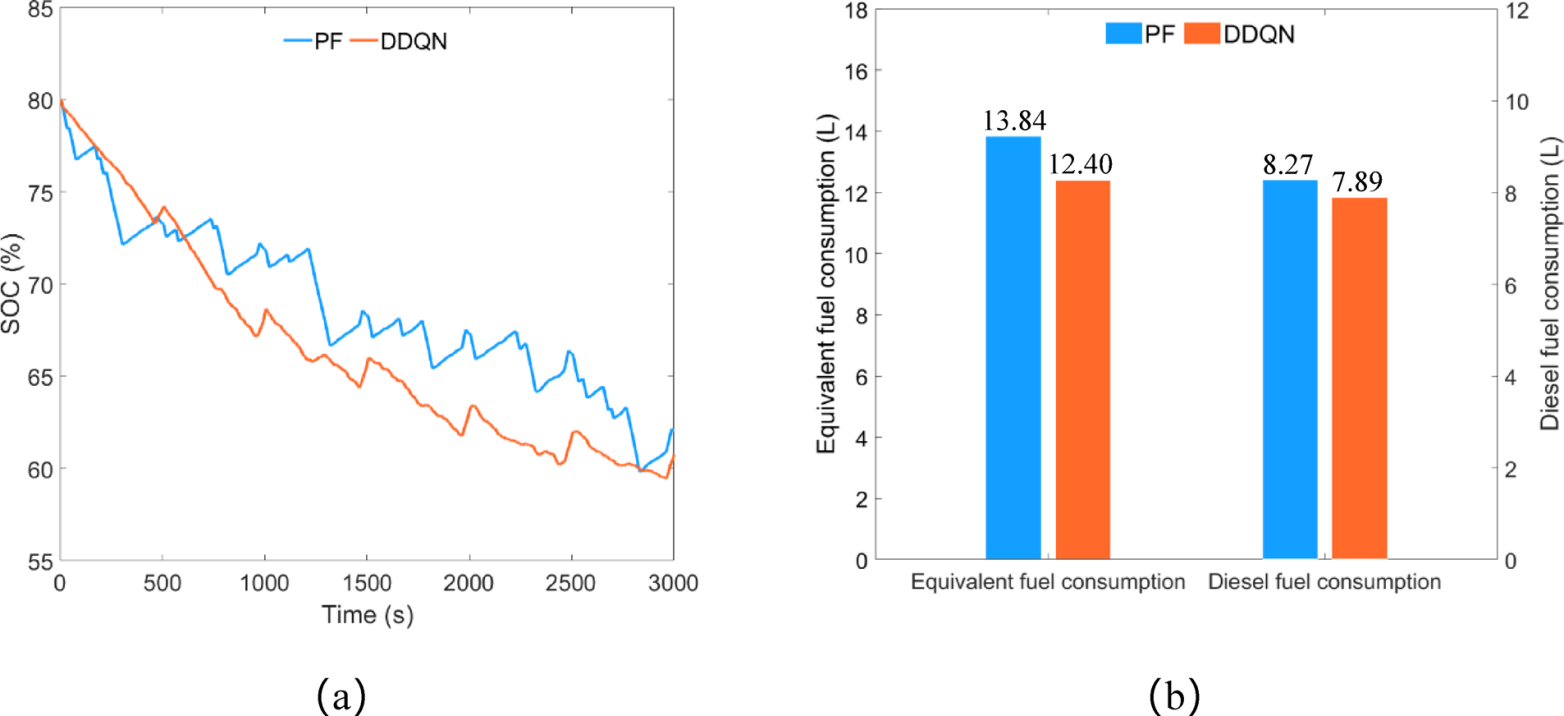
Plowing condition, (**a**) SOC change curve; (**b**) fuel consumption.

As shown in Fig. 14a, under the control of the PF strategy, the remaining SOC is approximately 60.04%, while under the control of the DDQN strategy, the remaining SOC is about 60.75%. The difference in remaining SOC between the two strategies is approximately 1.18%. Under the control of the DDQN strategy, the trend of the SOC curve is smoother compared to the PF strategy, with no continuous significant increases or decreases in SOC. As illustrated in Fig. 14b, the equivalent fuel consumption of the PF strategy and the DDQN strategy is approximately 13.84 L and 12.40 L respectively, and the diesel consumption is approximately 8.27 L and 7.89 L respectively. Compared to the PF strategy, the DDQN strategy reduces equivalent fuel consumption by 10.40% and diesel consumption by 4.59%.

#### Rotary tillage condition

**Fig. 15:**
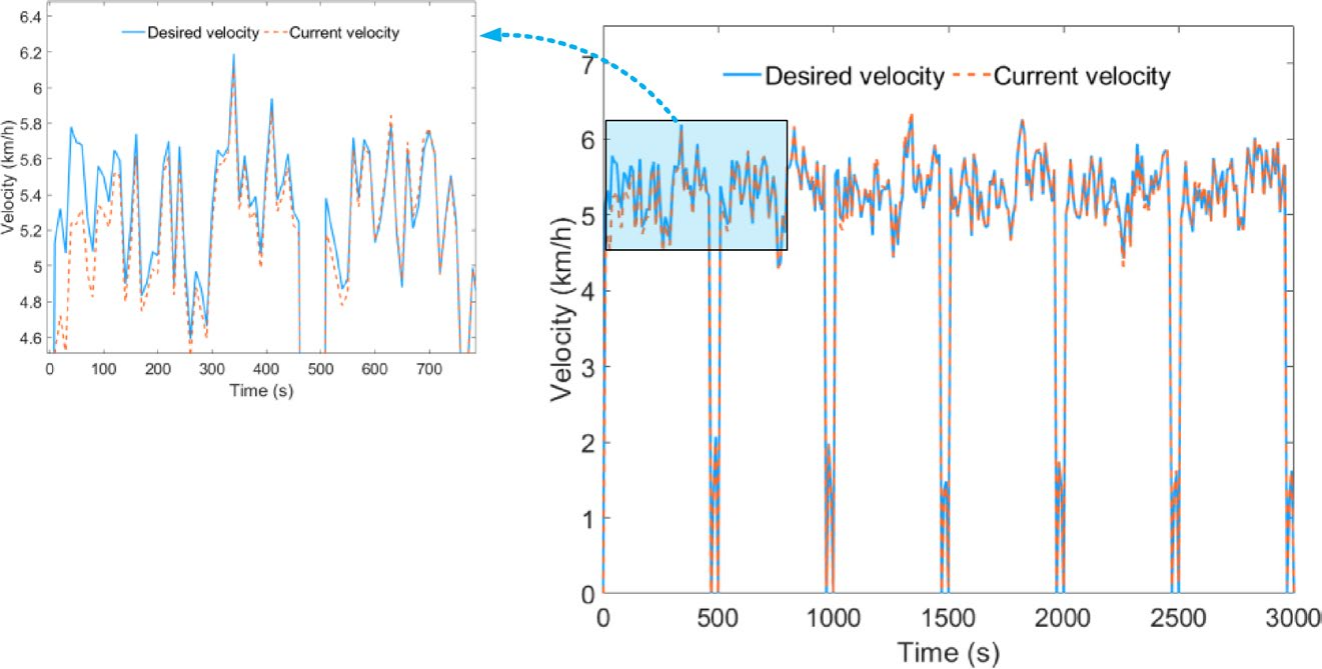
Vehicle velocity tracking effect under rotary tillage condition.

Under the rotary tillage condition, the desired vehicle velocity tracking effect of the HIL test is shown in Fig. 15, where the average error between the expected vehicle speed and the current vehicle speed remains at 0.030 km/h.

**Fig. 16:**
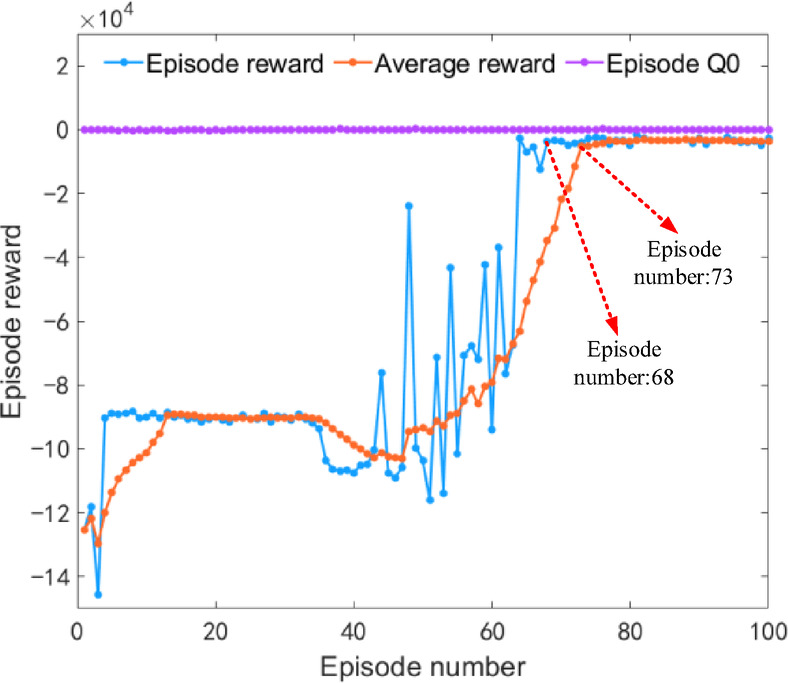
Iteration results of the DDQN algorithm under rotary tillage condition.

Under rotary tillage conditions, the iteration results of the DDQN algorithm are shown in Fig. 16. The iteration reward and average iteration reward began to converge at the 68th and 73rd iteration training processes, respectively. After 100 iteration trainings, the reward curve stabilized, verifying the reliability of the algorithm’s iteration results.

**Fig. 17:**
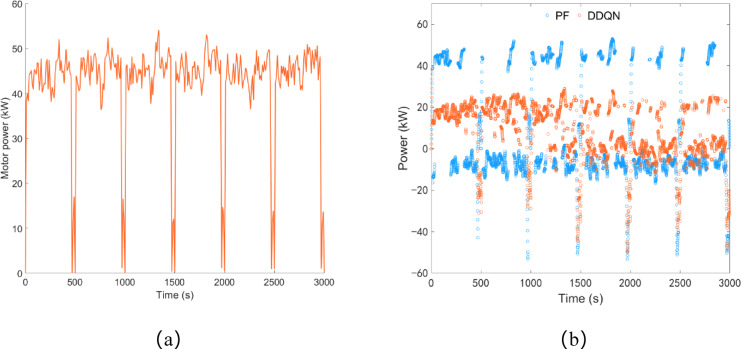
Rotary tillage condition, (**a**) drive motor power; (**b**) battery power.

As shown in Fig. 17a, under rotary tillage conditions, the driving motor power is concentrated between 40 and 50 kW, with an average load power of 42.10 kW, and the total power consumption is approximately 35.09 kWh. As shown in Fig. 17b, under the control of the DDQN strategy, the battery power variation is similar to that in plowing conditions. Approximately 11 s after the engine starts, it charges the battery, and thereafter the battery power remains below 29.08 kW. Similarly, under the control of the PF strategy, there are also multiple instances where the battery power is equal to the driving motor power.

**Fig. 18:**
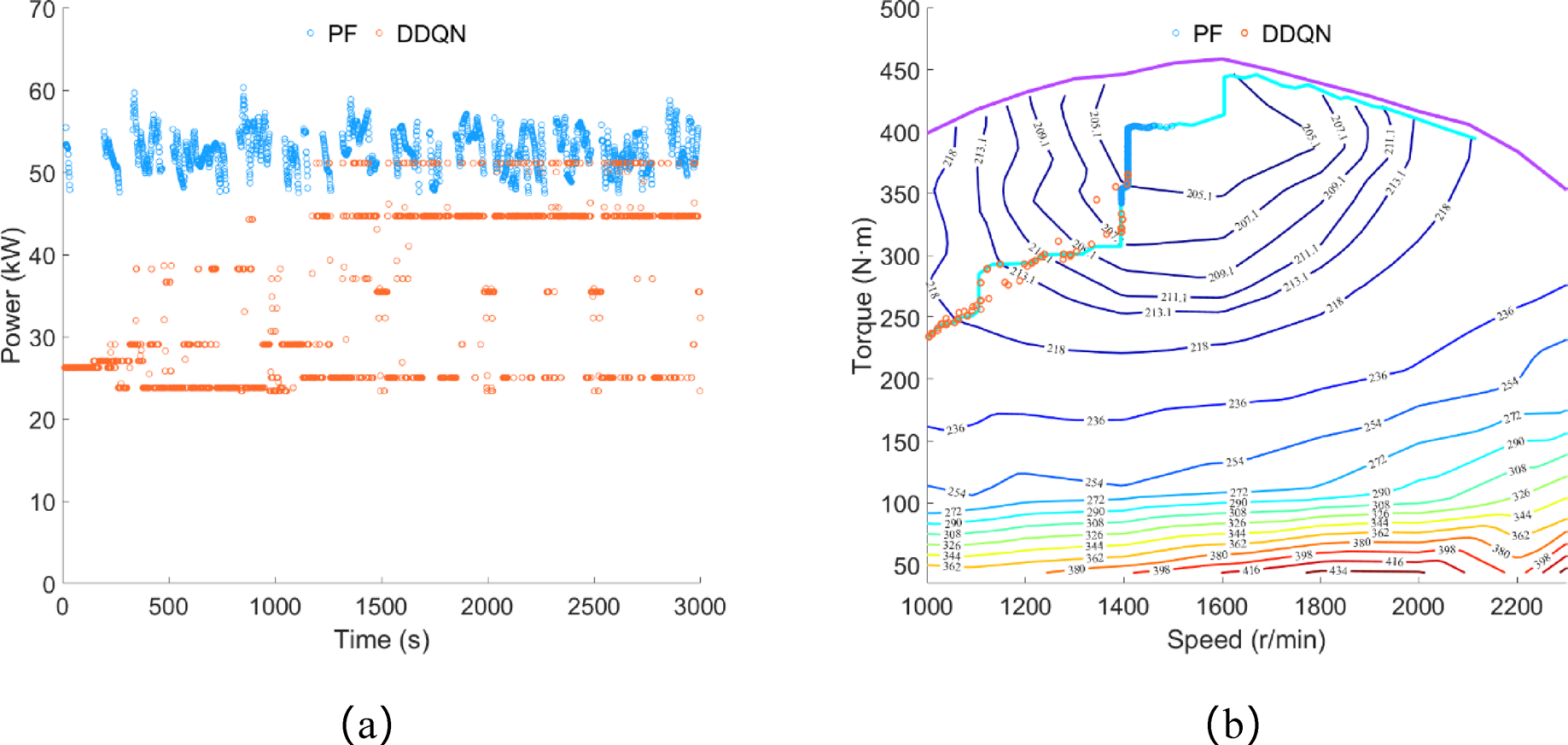
Rotary tillage condition, (**a**) engine power; (**b**) engine operating point.

As shown in Fig. 18a, under the control of the DDQN strategy, the engine power is concentrated between 23 and 51 kW, with an average power of 34.17 kW, and the total power consumption for electricity generation is approximately 28.48 kWh. Under the control of the PF strategy, the engine power is concentrated between 47 and 60 kW, with an average power of 35.04 kW, and the total power consumption for electricity generation is approximately 29.21 kWh. As shown in Fig. 18b, the engine operating points selected by both the PF strategy and the DDQN strategy are concentrated near the OOL curve. Similar to the results obtained in plowing conditions, the engine operating range is wider under the control of the DDQN strategy.

**Fig. 19:**
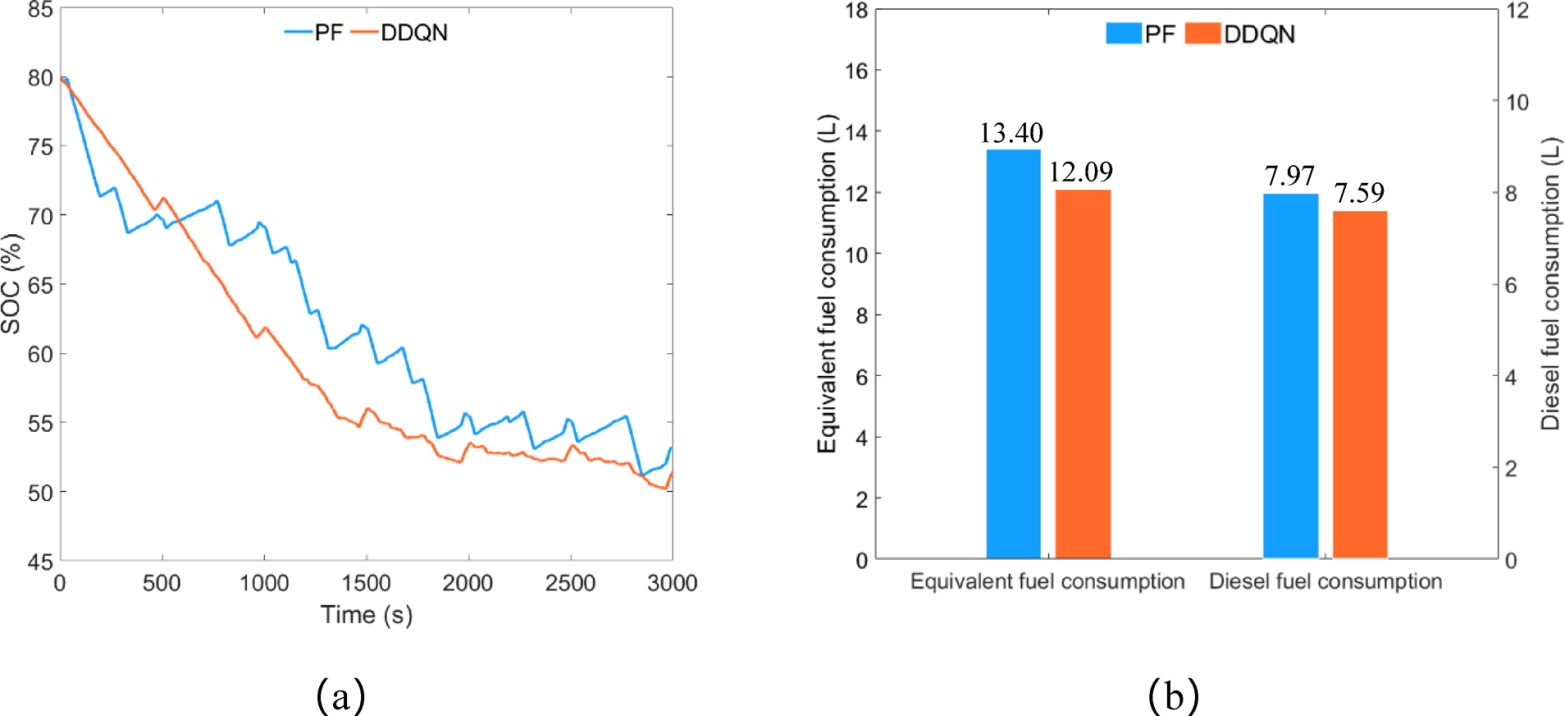
Rotary tillage condition, (**a**) SOC change curve; (**b**) fuel consumption.

As shown in Fig. 19a, the remaining SOC under the PF strategy control is approximately 53.1%, while under the DDQN strategy control, it is approximately 51.46%, with a difference of about 3.09% between the two strategies. Similar to the results obtained in plowing conditions, the trend of the SOC curve under the DDQN strategy control is gentler compared to the PF strategy. As shown in Fig. 19b, the equivalent fuel consumption under the PF strategy and DDQN strategy is approximately 13.40 L and 12.09 L respectively, with diesel consumption of approximately 7.97 L and 7.59 L respectively. Compared to the PF strategy, the DDQN strategy reduces equivalent fuel consumption by 9.78% and diesel consumption by 4.77%.

#### Transportation condition

**Fig. 20:**
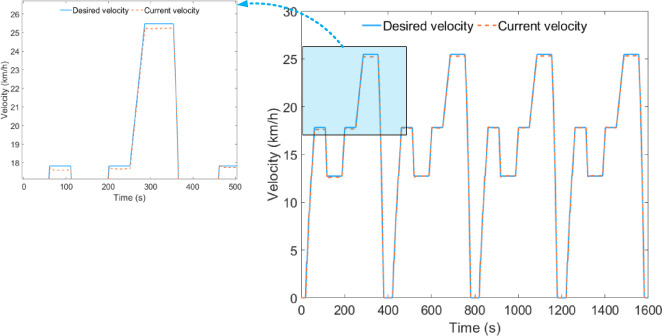
Vehicle velocity tracking effect under transportation condition.

Under transportation conditions, the desired vehicle velocity tracking performance during HIL testing is shown in Fig. 20, with the average error between the expected vehicle speed and the current vehicle speed maintained at 0.070 km/h.

**Fig. 21:**
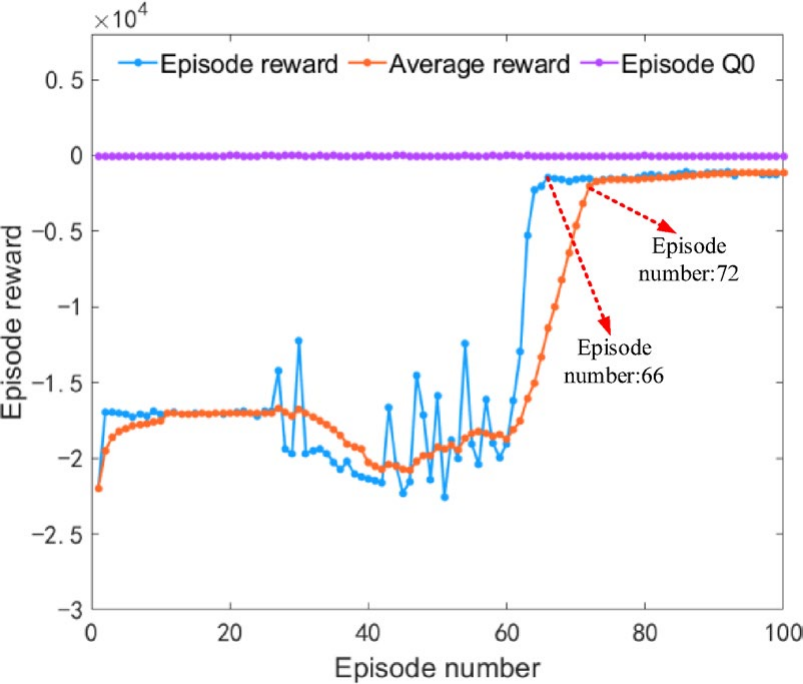
Iteration results of the DDQN algorithm under transportation condition.

Under transportation conditions, the iterative results of the DDQN algorithm are shown in Fig. 21. The iterative rewards and average iterative rewards begin to converge during the 66th and 72nd iterative training sessions respectively. After 100 iterations of training, the reward curve tends to stabilize, verifying the reliability of the algorithm’s iterative results.

**Fig. 22:**
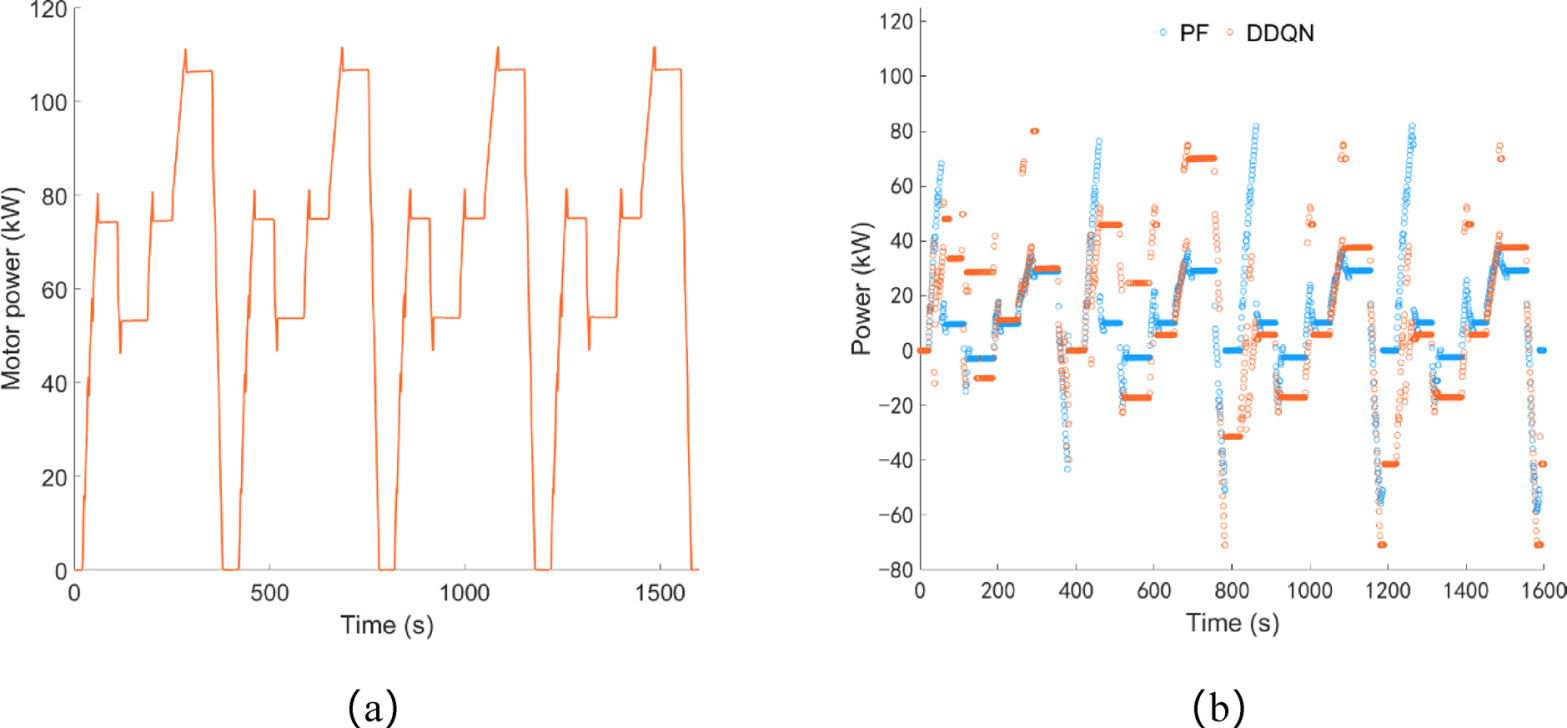
Transportation condition, (**a**) drive motor power; (**b**) battery power.

As shown in Fig. 22a, under transportation conditions, the driving motor power is concentrated between 50 and 105 kW, with an average load power of 65.63 kW, and the total power consumption is approximately 29.19 kWh. As shown in Fig. 22b, under the control of both the DDQN strategy and the PF strategy, the maximum charging power of the battery is 70.92 kW and 58.90 kW respectively, and the trend of battery power change is basically consistent.

**Fig. 23:**
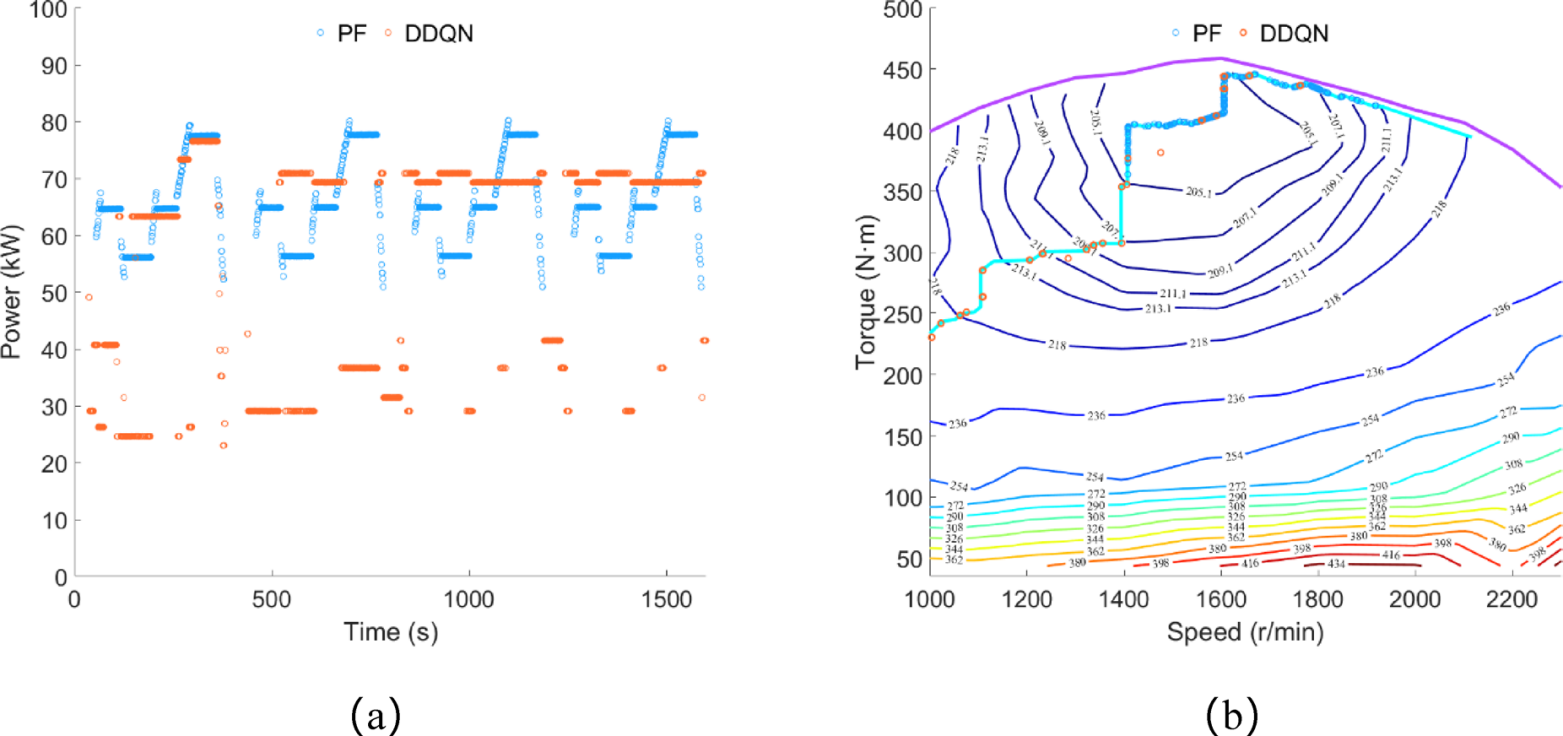
Transportation condition, (**a**) engine power; (**b**) engine operating point.

As shown in Fig. 23a, under the control of the DDQN strategy, the engine power is concentrated between 23 and 76 kW, with an average power of 53.61 kW, and the total power consumption for electricity generation is approximately 23.84 kWh. Under the control of the PF strategy, the engine power is concentrated between 50 and 80 kW, with an average power of 53.48 kW, and the total power consumption for electricity generation is approximately 23.78 kWh. As shown in Fig. 23b, the engine operating points selected by both the PF strategy and the DDQN strategy are concentrated near the OOL curve. Compared to the plowing and rotary tillage conditions, the engine operating range under both control strategies has increased.

**Fig. 24:**
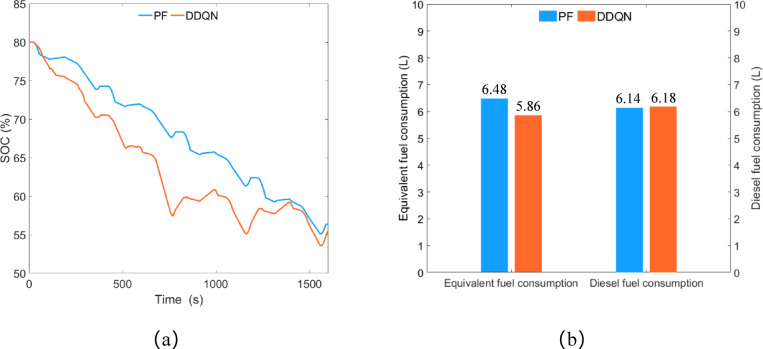
Transportation condition, (**a**) SOC change curve; (**b**) fuel consumption.

As shown in Fig. 24a, the remaining SOC under the PF strategy control is approximately 56.35%, while under the DDQN strategy control, it is approximately 55.51%, with a difference of about 1.49% between the two strategies. As shown in Fig. 24b, the equivalent fuel consumption under the PF strategy and DDQN strategy is approximately 6.48 L and 5.86 L respectively, with diesel consumption of approximately 6.14 L and 6.18 L respectively. Compared to the PF strategy, the DDQN strategy reduces equivalent fuel consumption by 9.57% but increases diesel consumption by 0.65%.

### Comparative discussion

**Fig. 25:**
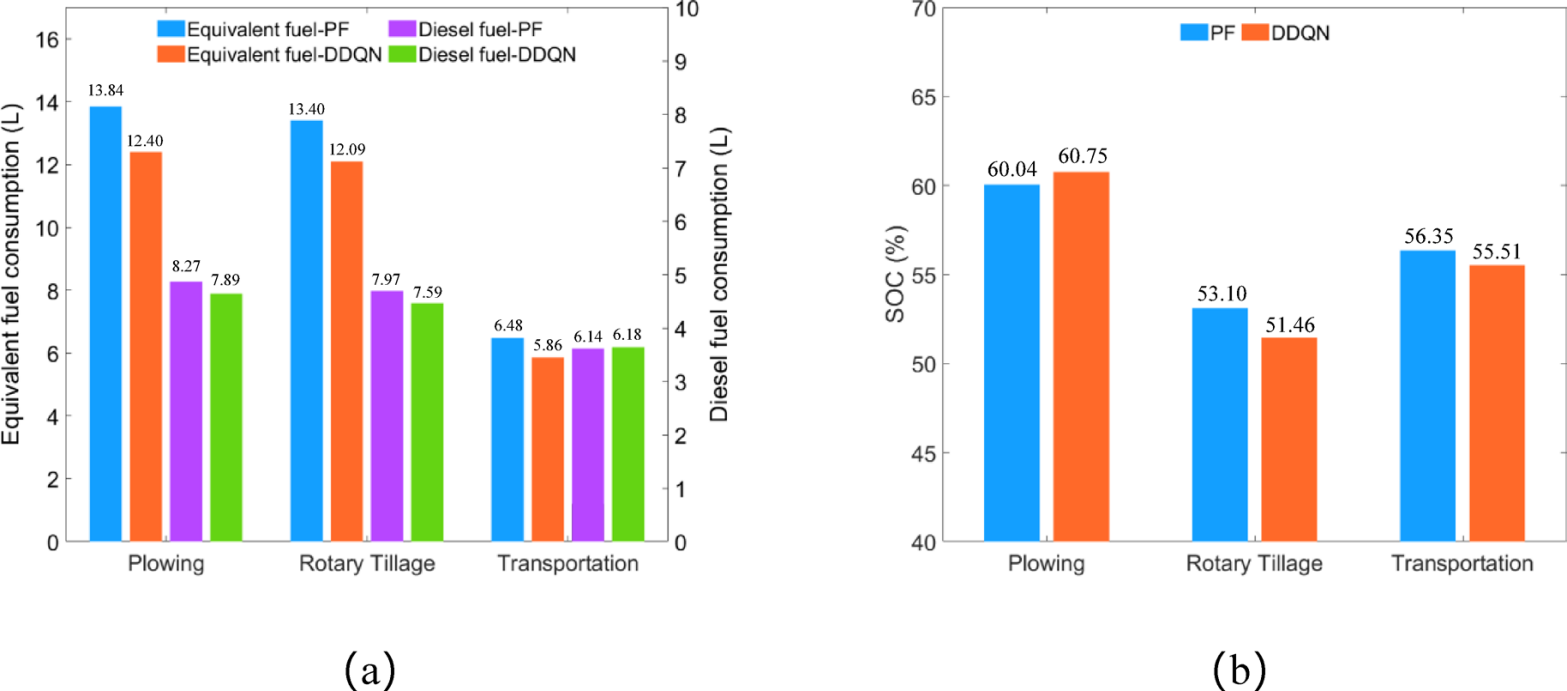
HIL test results, (**a**) equivalent fuel consumption and diesel consumption; (**b**) remaining SOC.

The HIL test results for the DDQN strategy and PF strategy under different operating conditions are shown in Fig. 25.

Under plowing conditions, the DDQN strategy saved 1.18% of SOC, reduced equivalent fuel consumption by 10.40%, and decreased diesel consumption by 4.59% compared to the PF strategy.

Under rotary tillage conditions, the DDQN strategy consumed 3.09% more SOC, reduced equivalent fuel consumption by 9.78%, and decreased diesel consumption by 4.77% compared to the PF strategy.

Under transportation conditions, the DDQN strategy consumed 1.49% more SOC, reduced equivalent fuel consumption by 9.57%, and increased diesel consumption by 0.65% compared to the PF strategy.

Based on the analysis of the HIL test data results, it can be concluded that under the three operating conditions of plowing, rotary tillage, and transportation, the DDQN strategy consumes up to 3.09% more remaining SOC and increases diesel consumption by a maximum of 0.65% compared to the PF strategy. Therefore, it can be inferred that under the premise of similar energy consumption, the DDQN strategy significantly reduces equivalent fuel consumption compared to the PF strategy.

## Conclusions

To further optimize the fuel economy of high-power diesel-electric hybrid tractors, an energy management strategy based on DRL is proposed, using equivalent fuel consumption as the optimization objective and based on the Double Deep Q-Network algorithm. The main conclusions of this study are as follows:

To validate the proposed energy management strategy, a HIL testing platform was set up, consisting of Matlab/Simulink, the PowerECU-57A vehicle ECU controller, and a HIL testing cabinet. HIL testing was conducted on the simulation model and energy management strategy developed in this study. The test results indicate that during the HIL testing process, the average error between the desired vehicle speed and the current vehicle speed remained within the range of 0.028–0.070 km/h, effectively validating the effectiveness of the simulation model and control strategy.

Under plowing and rotary tillage conditions, the DDQN strategy is able to better control the engine operating state and output power compared to the PF strategy. While avoiding frequent engine start–stops, the trend of the SOC curve is also gentler compared to the PF strategy, without continuous large fluctuations in SOC. This effectively prolongs the service life of the traction battery.

The HIL test results indicate that, compared to the PF strategy, the DDQN strategy: under plowing conditions, saves 1.18% of SOC , reduces equivalent fuel consumption by 10.40%, and decreases diesel consumption by 4.59%; under rotary tillage conditions, consumes 3.09% more SOC, reduces equivalent fuel consumption by 9.78%, and decreases diesel consumption by 4.77%; and under transportation conditions, consumes 1.49% more SOC, reduces equivalent fuel consumption by 9.57%, and increases diesel consumption by 0.65%. With little difference in remaining SOC and diesel consumption, the DDQN strategy significantly reduces equivalent fuel consumption and improves economy compared to the PF strategy.

At present, preliminary progress has been made in the research field of energy management strategies for hybrid electric tractors based on DRL. DRL is an important direction for energy-saving optimization control of hybrid electric tractors. In subsequent research, more advanced DRL algorithms, such as DDPG algorithm, Dueling DQN algorithm, and multi-agent reinforcement learning, should be considered. Meanwhile, different operating conditions have a significant impact on the energy-saving effects of energy management strategies for hybrid electric tractors. This paper conducted a comparative analysis of three typical operating conditions of tractors, but it was not comprehensive enough. Taking plowing conditions as an example, different operating regions, such as plains, hills, and mountains, as well as different farming seasons, have a substantial influence on the load variations of tractor operating conditions. In subsequent research, we will attempt to consider the impact of subdivided operating conditions on energy management strategies.

## Data Availability

The original contributions presented in the study are included in the article; further inquiries can be directed to the corresponding author.
